# Molecular basis for SOX2-dependent regulation of super-enhancer activity

**DOI:** 10.1093/nar/gkad908

**Published:** 2023-11-01

**Authors:** Wanki Yoo, Yi Wei Song, Jihyun Kim, Jihye Ahn, Jaehoon Kim, Yongdae Shin, Je-Kyung Ryu, Kyeong Kyu Kim

**Affiliations:** Department of Precision Medicine, Graduate School of Basic Medical Science (GSBMS), Institute for Antimicrobial Resistance Research and Therapeutics, Sungkyunkwan University School of Medicine, Suwon 16419, Republic of Korea; Department of Precision Medicine, Graduate School of Basic Medical Science (GSBMS), Institute for Antimicrobial Resistance Research and Therapeutics, Sungkyunkwan University School of Medicine, Suwon 16419, Republic of Korea; Department of Biological Sciences, Korea Advanced Institute of Science and Technology, Daejeon 34141, Republic of Korea; Department of Biological Sciences, Korea Advanced Institute of Science and Technology, Daejeon 34141, Republic of Korea; Department of Biological Sciences, Korea Advanced Institute of Science and Technology, Daejeon 34141, Republic of Korea; Department of Mechanical Engineering, Seoul National University, Seoul 08826, Republic of Korea; Department of Physics & Astronomy, Seoul National University, Seoul 08826, Republic of Korea; Department of Precision Medicine, Graduate School of Basic Medical Science (GSBMS), Institute for Antimicrobial Resistance Research and Therapeutics, Sungkyunkwan University School of Medicine, Suwon 16419, Republic of Korea

## Abstract

Pioneer transcription factors (TFs) like SOX2 are vital for stemness and cancer through enhancing gene expression within transcriptional condensates formed with coactivators, RNAs and mediators on super-enhancers (SEs). Despite their importance, how these factors work together for transcriptional condensation and activation remains unclear. SOX2, a pioneer TF found in SEs of pluripotent and cancer stem cells, initiates SE-mediated transcription by binding to nucleosomes, though the mechanism isn’t fully understood. To address SOX2’s role in SEs, we identified mSE078 as a model SOX2-enriched SE and p300 as a coactivator through bioinformatic analysis. In vitro and cell assays showed SOX2 forms condensates with p300 and SOX2-binding motifs in mSE078. We further proved that SOX2 condensation is highly correlated with mSE078’s enhancer activity in cells. Moreover, we successfully demonstrated that p300 not only elevated transcriptional activity but also triggered chromatin acetylation via its direct interaction with SOX2 within these transcriptional condensates. Finally, our validation of SOX2-enriched SEs showcased their contribution to target gene expression in both stem cells and cancer cells. In its entirety, this study imparts valuable mechanistic insights into the collaborative interplay of SOX2 and its coactivator p300, shedding light on the regulation of transcriptional condensation and activation within SOX2-enriched SEs.

## Graphical Abstract

**Graphical Abstract:**
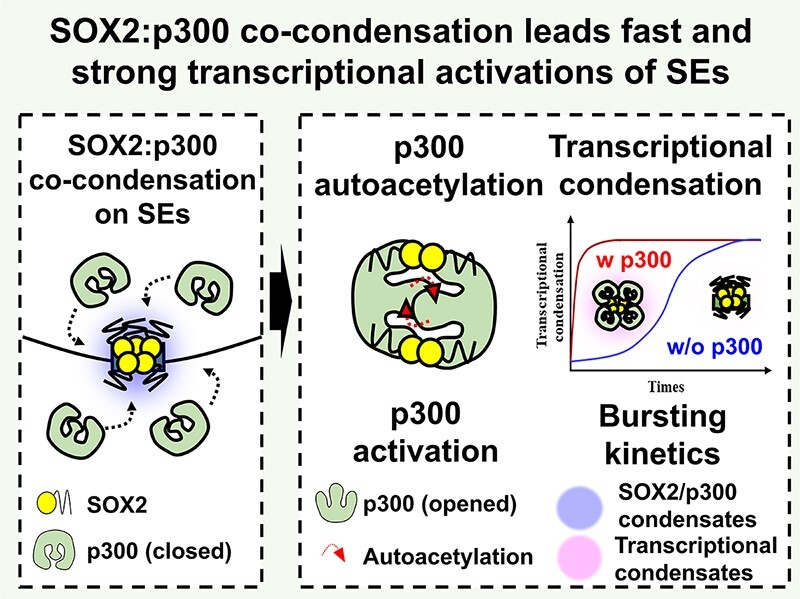

## Introduction

Enhancers are responsible for long-range gene expression control, whereas promoters located near the transcription start site directly affect gene expression as c*is*-regulatory elements (1). Enhancer activity is regulated by various *trans*-regulatory elements such as transcription factors (TFs), coactivators and mediators (2–8). Super-enhancers (SEs) are defined as regions comprising single or multiple enhancers. They differentiate from typical enhancers. SEs are highly associated with the expression of cell-identity genes and heavily bind to mediators, pioneer TFs and coactivators in the genome (9). Therefore, active sets of SEs can dynamically switch during lineage selection, progression and cellular senescence (10,11). Accordingly, precise control of transcriptional activation of SEs in response to environmental cues is critical for maintaining cell differentiation and stability. In contrast, aberrant regulation of SE activity leads to severe diseases such as cancer (12–15).

Among SE-associated factors involved in the dynamic regulation of transcriptional activation of SEs, pioneer TFs play key roles in determining a repertoire of SEs in a cell type- or condition-specific manner by initiating chromatin opening through direct interaction with nucleosomes (10,16). Binding of pioneer TFs to nucleosomes triggers disruption of local chromatin structure, which is necessary for recruiting SE-associated coactivators involved in the modification of histones and DNA (3,17–19). The mechanism of SE-mediated gene regulation can be explained in several ways (20–22). Among these, a phase separation model can explain the enrichment of components of SEs and subsequent transcriptional activation, which is consistent with experimental evidence (23).

In the phase-separation model, transcriptional components enriched in SEs are proposed to contribute to the formation of transcriptional condensates, which are dynamically regulated by various factors such as interactions between TFs and coactivators, local chromatin architectures, concentrations of components and sequences and epigenetic features of SEs in the genome (24–28). Functionally, formation of transcriptional condensates is implicated in robust transcriptional activation of SEs because of the following events in condensates: rearrangement in a local chromatin structure, fast formation of transcription initiation component and co-condensation with RNA polymerase II (29–34). As pioneer TFs are upstream factors for the initiation of SE-dependent transcriptional activation, they play a key role in initiating transcriptional condensation at specific loci of SEs depending on the number, density, affinity and modification of TF binding motifs (25,28). In addition, co-condensation of pioneer TFs with coactivators, mediators and other TFs is known to influence transcriptional condensation and activation (24,28,29,34).

Among pioneer TFs in stem cells, OCT4, KLF4 and SOX2 have been revealed to form condensates with DNAs encoding their cognate TF binding motifs (25,28,35). Although co-condensation factors and the mechanism of TF condensation with DNA have been well studied for OCT4 and KLF4, those for SOX2, a pioneer TF enriched at stem cells-SEs and an upstream factor in cellular reprogramming to pluripotent stem cells, are relatively less elucidated (24,25,28). For example, SOX2 condensation has been tested using bacteriophage λ DNA as a model DNA instead of the natural target counterpart (35). Furthermore, co-condensation of SOX2 with a coactivator has only been demonstrated using the transactivation domain (TAD) of SOX2 in the absence of DNA (24,34). Accordingly, the mechanism of SOX2 condensation with cofactors on DNA and functional implications of SOX2 condensation in transcriptional activation remain unclear. Therefore, contribution of SOX2 and its associated molecules to transcriptional condensation and activation of SEs and their controls of SE-mediated gene expression remain unclear. Therefore, we first identified mSE078 as a model for SOX2-enriched SE. It could function as an enhancer of *MKRN1*, an E3 ubiquitin ligase known to regulate metabolic diseases and malignancies. We also found that p300 was a potential SOX2 coactivator at mSE078 based on publicly available SOX2 ChIP-seq data. Our results demonstrated that SOX2 could condense with mSE078 in a motif-dependent manner. Furthermore, using bio-layer interferometric (BLI) analysis, *in vitro* acetylation assay, luciferase reporter assay and confocal microscopic analysis, we verified that p300 could lead to a strong and fast transcriptional condensation and activation by direct interaction with SOX2 through its KIX domain. Finally, we confirmed the implication of SOX2-enriched SE in MKRN1 expression of cancer stem cells. Taken together, these findings provide a comprehensive understanding of the mechanism by which SOX2 and its coactivator p300 cooperatively regulate SE-mediated gene expression via transcriptional condensation, which can be a potential therapeutic target for treating cancer.

## Materials and methods

### Genomic analysis and preparation of luciferase reporter plasmids

Super-enhancer annotations for mouse embryonic stem cells (mESCs) were retrieved from a previous study (9). Publicly available ChIP-seq data for SOX2, OCT4, NANOG and MED1 in mESCs were downloaded from Gene Expression Omnibus (GEO) database (GSE44288). H3K27ac ChIP-seq data for mouse embryonic fibroblast (MEF) cells were downloaded from the GEO database (GSM851277). ChIP-seq data for SOX2 and H3K27ac and RNA-seq data for KYSE-70 cells were downloaded from the GEO database (GSE167366 and GSE167365). ChIP-seq data for RNA Pol II were downloaded from the GEO database (GSM5722878). All other ChIP-seq data for p300, MED1, H3K27ac and CTCF and RNA-seq data for mESCs, MEF cells and hESCs were downloaded from Integrative Genomics Viewer (36). RNA Pol II Chromatin interaction assay paired-end tag (ChIA-PET) data for mESCs and HeLa S3 cells were downloaded from the GEO database (GSM4041604 and GSM970211, respectively). Images for ChIP-seq data were prepared using Integrative Genomics Viewer.

The genomic region of mSE078 (Chr6:mm9:39395572–39396779, 1208 bp) was synthesized and further dissected into enhancer fragments based on SOX2 ChIP-seq signals. The region (Chr6:mm9:39395945–39396116, 172 bp) showing the highest SOX2 signal was annotated as SM. The region (Chr6:mm9:39396430–39396601, 172 bp) showing the lowest SOX2 signal was annotated as NM. There was only one SOX2 binding consensus motif (5′-CTTTTGT-3′, Chr6:mm9:39396022–39396028) in SM. It was substituted with 5′-CTTATCT-3′ using a Quik-change polymerase chain reaction (PCR) method to generate a defective SOX2 binding motif (dSM) (37). A putative core promoter region (Chr6:mm9:39420541–39420742, 201 bp) of the mouse *MKRN1* gene was synthesized and cloned into the pGL3-Basic reporter plasmid using HindIII and XhoI sites at the 5′ and 3′ ends, respectively. DNA fragments of mSE078 (SM, NM, or dSM) were cloned into the pGL3-*MKRN1* reporter plasmid using BamHI and SalI sites at 5′ and 3′ ends, respectively. For luciferase reporter plasmid for OE33 cells and HEK cells, a human *MKRN1* core promoter (chr7:hg19:140178868–140179367, 500 bp) and a putative human *MKRN1* SE (-5SE, chr7:hg19:140185361–140185675, 315 bp) were synthesized and cloned into pGL3-Basic plasmid. The core promoter regions were chosen based on binding of RNA Pol II. All restriction enzymes and T4 DNA ligase were purchased from New England Biolabs (Ipswich, MA, USA). Gel extraction, plasmid purification and PCR purification kits were obtained from Cosmogenetech (Seoul, Korea).

### Cloning and purification of proteins

The DNA encoding the full-length human SOX2 (uniport ID: P48431, 317 amino acids) was cloned into a pVFT1S plasmid using NheI and XhoI sites at 5′ and 3′ ends, respectively. Since an N-terminal His-tag peptide (MGHHHHHHAS) was included for the purification purpose in the bacterial expression plasmid (pVFT1S plasmid), the recombinant SOX2 used in this study contains 327 amino acids. The recombinant plasmid was transformed into *Escherichia coli* BL21(DE3), which was then cultured at 37°C to an optical density at 600 nm (OD_600 nm_) of 0.5 to 0.6 in Luria-Bertani (LB) medium containing 50 mg/l kanamycin. After protein induction by isopropyl-*β*-d-thiogalactopyranoside (IPTG) at 37°C for 4 h, cells were harvested and lysed by sonication (420 cycles of on for 1 s and off for 3 s). Cell lysates were centrifuged at 40 000 g for 1 h. Cell pellets were then resuspended in a denaturing buffer (20 mM HEPES–NaOH, 500 mM NaCl, 8 M urea, 20 mM imidazole and 10 mM *β*-mercaptoethanol, pH 7.3) for 2 h. After centrifugation at 40 000 g for 1 h, the supernatant was loaded onto a HisTrap HP column (Cytiva, Marlborough, MA, USA), washed with denaturing buffer and eluted using a denaturing buffer containing 500 mM imidazole. Refolding of SOX2 was achieved by diluting with a refolding buffer (20 mM HEPES–NaOH, 300 mM l-arginine, 100 mM KCl and 5 mM MgCl_2_, pH 7.3) and incubating at 16°C for 16 h (38). Correctly folded SOX2 was further purified using a Superose 6 Increase 10/300 column (Cytiva, Marlborough, MA, USA) equilibrated with 20 mM HEPES–NaOH, 100 mM KCl, 50 mM arginine and 5 mM MgCl_2,_ pH 7.3 on AKTA pure (Cytiva, Marlborough, MA, USA).

DNA-binding domain of SOX2 (SOX2_DBD_, aa 39–127), C-terminal domain of SOX2 (SOX2_CTD_, aa 150–317) and human p300 fragments [KIX, aa 566–646; Bromodomain (BD), aa 1067–1139; KIX-BD (KB), aa 566–1139; and BD-HAT (BH), aa 1067–1663) were cloned into the pVFT1S plasmid using NheI and XhoI sites at 5′ and 3′ ends, respectively. After sequencing, recombinant cells were prepared in the same manner as for SOX2, but purified from supernatants after sonication and centrifugation. Supernatants were loaded onto a HisTrap HP column, washed and eluted with an imidazole gradient of 20–500 mM. All proteins were further purified using a Superose 6 Increase 10/300 column equilibrated with 20 mM HEPES–NaOH, 100 mM KCl and 5 mM MgCl_2,_ pH 7.3. The purity of proteins was checked by SDS-PAGE. Their concentrations were determined by BCA assay using BSA as a standard protein.

For expression in mammalian cells, human and mouse SOX2, OCT4 and NANOG were cloned into pcDNA3.1 plasmid using XbaI and KpnI at 5′ and 3′ ends, respectively. Plasmids expressing mutant SOX2 were generated using the QuikChange PCR method (39). All primers used in this study are listed in Supplementary Table S1.

### Electrophoretic mobility shift assay (EMSA)

Binding of SOX2 and SOX2_DBD_ to SM or nucleosomal SM (NuSM) was monitored using EMSA. Binding reactions were carried out in 5 μl (total volume) binding buffer (10 mM HEPES–NaOH, pH 7.3, 50 mM KCl, 5 mM MgCl_2_, 0.5 mM DTT, 5% glycerol and 0.5 mg/ml BSA). Binding of p300 (Protein One, Gaithersburg, MD, USA) and p300 fragments on SOX2 bound-SM or -NuSM were investigated by adding p300 or p300 fragments to the above mixtures. After 1 h of incubation, samples were run on 4% non-denaturing polyacrylamide gel in 0.5× TBE buffer at 70 V for 60 min at 4°C and visualized by staining with SYBR Gold staining dye (Thermo Fisher Scientific, Waltham, MA, USA). Images were obtained using ChemiDoc XRS^+^ and analyzed with ImageLab software (Bio-Rad, Hercules, CA, USA). All experiments were performed at least twice. DNA amount was quantified based on SYBR Gold fluorescence. Total amount of DNA was quantified from free DNA band at 0 nM SOX2_DBD_ concentration. Binding fraction was calculated with the following formula:


\begin{eqnarray*}{\mathrm{Binding\ fraction\ of\ }}{{\rm DNA}}_{{\rm bound}}\ \left( {\mathrm{\% }} \right) &=& \left[ {1 - \left( {\frac{{{{\rm DNA}}_{free}}}{{{{\rm DNA}}_{{\rm total}}}}} \right)} \right] \\ && \times 100\end{eqnarray*}

Fractions of bound DNA as a function of SOX2_DBD_ condensation from two separate experiments were fitted to the Hill equation using nonlinear regression in GraphPad Prism software. Apparent *K*_D_ of SOX2_DBD_ to each DNA element was calculated by fixing *B*_max_ to 1:


\begin{eqnarray*}{\mathrm{Binding\ fraction\ of\ }}{{\rm DNA}}_{{\rm bound}} = \ \frac{{{B}_{{\rm max}}\ \times \ {X}^h}}{{{K}_D^h + \ {X}^h}}\end{eqnarray*}

where *B*_max_ was the maximum binding fraction of DNA_bound_, *X* was the concentration of proteins, and *h* was the Hill coefficient.

### Chromatin immunoprecipitation and quantitative real-time PCR (ChIP-qPCR)

For ChIP-qPCR analysis with SOX2 knockdown or expression of mutant SOX2, mESCs (R1 cell line, which is F1 hybrid between 129/Sv and 129/SvJ) or MEF (NIH-3T3) cells were subcultured in 24-well plates for one day before transfection. On the day of transfection, SOX2 siRNA and negative control siRNA (S13294; Invitrogen, Waltham, MA, USA), which were pre-incubated for 5 min with HiPerFect reagent (QIAGEN, Hilden, Germany), were transiently transfected into mESCs. pcDNA3.1 with mutant SOX2, which was pre-incubated with Turbofect (Thermo Fisher Scientific, Waltham, MA, USA) for 20 min, was transiently transfected into MEF cells.

After incubating at 37°C in a CO_2_ incubator for 1 day, cells were fixed with 1% formaldehyde, stopped by glycine buffer, scrapped in PBS with 0.5 mM PMSF and centrifuged at 1000 rpm for 10 min at 4°C. After cell lysis and homogenization, nuclei were digested and chromatins were sheared with an enzyme cocktail (Active motif #53014, Carlsbad, CA, USA). Sheared chromatin was incubated with specified antibody and protein G magnetic beads for 16 h at 4°C. These beads were pulled down, washed and incubated in elution and reverse-crosslinking buffers at 95°C for 15 min. After incubating with proteinase K for 1 h at 37°C, the solution was used directly for qPCR. qPCR was performed using an iTaq Universal SYBR Green Supermix (Bio-Rad, Hercules, CA, USA). Primers used for qRT-PCR are listed in Supplementary Table S1.

### *In vitro* condensation assay

For SOX2 condensation, full-length SOX2 was labeled with Cy3-Maleimide (Click Chemistry Tools, Scottsdale, AZ, USA). SOX2-Cy3 (final concentration of 837 nM) was mixed with enhancer fragments in PS buffer (20 mM Tris–HCl, 80 mM NaCl, 5 mM MgCl_2_ and 10% PEG8000). After 1 h of incubation at room temperature, 3 μl of the sample was loaded onto a glass slide, covered by a cover slip and observed under a fluorescence microscope. To assess the effect of p300 on SOX2 condensation, SOX2-Cy3 (final concentration 558 nM) and SM (final concentration 100 nM) were mixed with different concentrations (0–126 nM) of p300 in PS buffer. After incubation for 10 min at room temperature, samples were observed as described above. For co-condensation of SOX2 and p300, full-length p300 was labelled with AF430-Maleimide (Lumiprobe, Hunt Valley, MD, USA). SOX2-Cy3, SM and p300-AF430 (final concentration: 126 nM) mixed in PS buffer and imaged after incubation for 1 h at room temperature.

To quantify droplet formation, five randomly chosen regions were visualized from each sample at the same exposure time and ISO setting. Fluorescence intensities in images (TIF files) were determined using ImageJ (40). The intensity threshold was set at 100, which was the maximum intensity from images of SOX2 alone. Other settings were default. Fluorescent intensity, area, counts and size droplets were measured using the particle analysis tool in ImageJ software. Experiments were performed at least twice.

### Luciferase reporter assay

For the luciferase assay, mESCs (R1 cell line) and MEF (NIH-3T3) were cultured in 24-well plates for 1 day before transfection. On the day of transfection, cells were transiently transfected with 900 ng pGL3 plasmid and 100 ng pRL-TK (internal control) using Turbofect (Thermo Fisher Scientific, Waltham, MA, USA). For co-transfection of pcDNA3-mSOX2, 450 ng of pGL3 plasmid and 50 ng of pRL-TK were used for co-transfection with 500 ng of pcDNA3 plasmid at different ratios of pcDNA3:mSOX2 and empty pcDNA3 plasmid. One day after transfection, a luciferase assay was performed using a dual luciferase assay kit (Promega, Madison, WI, USA). All luciferase assays were performed at least in biological duplicates, starting from independent cell cultures. Luciferase assay for OE33 cells was performed as described above. Transfection was performed for trypsinized cells for 2 h.

### Bio-layer interferometric (BLI) analysis and pull-down assay

For BLI analysis, 0.2 mg/ml SOX2 was immobilized on an AR2G chip (Sartorius, Göttingen, Germany) and quenched by 1 M ethanolamine. Baseline was acquired using PBS. Various concentrations of p300 (0–400 nM) and KIX (0–43 μM) were dissociated in PBS. Equilibrium binding constant (*K*_D_), association rate constant (*k*_a_) and dissociation rate constant (*k*_d_) were calculated by global fitting using a 1:1 binding model with a BLItz Pro v.1.2.1.5 software (Sartorius, Göttingen, Germany). To determine *k*_a_ and *k*_d_, *k*_obs_ was calculated using the following equation:


\begin{eqnarray*}Y = {Y}_0 + A(1 - {{\rm e}}^{ - {k}_{{\rm obs}}*t)}\end{eqnarray*}

where *Y* was the level of binding (wavelength shift), *Y*_0_ was the binding at start of association, *A* was an asymptote, and *t* was the time. *k*_obs_ was the overall rate of the combined association and dissociation of two binding partners. Then *k*_d_ was calculated using the following equation:


\begin{eqnarray*}Y = Y_0 + A{{\rm e}}^{ - {k}_d*t}\end{eqnarray*}

where *Y*_0_ was binding at the start of dissociation. The following equation was then used to calculate *k*_a_:


\begin{eqnarray*}{k}_{\rm a} = \frac{{{k}_{{\rm obs}} - {k}_{\rm d}}}{{\left[ {{\rm Analyte}} \right]}}\end{eqnarray*}

Finally, *K*_D_, the affinity constant or equilibrium dissociation constant, was calculated using the following equation:


\begin{eqnarray*}{K}_{\rm D} = \frac{{{k}_{\rm d}}}{{{k}_{\rm a}}}\end{eqnarray*}

For the pull-down assay, 100 ng of biotinylated SM, 50 ng of SOX2 and 100 ng of p300 (Protein One, Gaithersburg, MD, USA) or p300 fragments were mixed with 5 μl (total volume) binding buffer containing 100 mM KCl and 0.5% NP-40. After incubation at 25°C for 1 h, streptavidin magnetic beads (Thermo Fisher Scientific, Waltham, MA, USA) pre-equilibrated in binding buffer were added followed by incubation at 25 °C for another 30 min. These beads were collected using magnets, washed with binding buffer, and resuspended in SDS sample buffer (50 mM Tris–HCl, pH 7.5, 2% SDS, 10% glycerol, 100 mM DTT and 0.005% bromophenol blue). Samples were then analyzed by western blotting. All experiments were performed in duplicate.

### Plasmid preparation for confocal microscopic analysis

Cellular localization and transcriptional activation of SOX2 and p300 in cells were analyzed using confocal microscopy. SOX2-T2A-mCherry plasmid was generously provided by Jennifer Mitchell (Addgene, #127538, Watertown, MA, USA). To create fusion constructs, p300 and EGFP were cloned into the SOX2-T2A-mCherry plasmid using XbaI (5′)/NheI (3′) restriction enzymes for p300 and KpnI (5′)/AgeI (3′) restriction enzymes for EGFP. To construct NLS-tdPCP-CFP, we replaced GFP with CFP using AgeI (5′)/NotI (3′) restriction enzymes in the NLS-tdPCP-GFP plasmid (Addgene, #183934). To generate pGL3-PP7-SE reporter plasmids, we amplified 12 × PP7 by PCR from pDZ617 pKAN 12 × PP7 (Addgene, #72237) and inserted it into pGL3-SM, dSM or NM plasmid using NcoI restriction enzyme.

### Confocal microscopic analysis

To analyze cellular localization of SOX2 and p300, mESCs (R1 cell line) and MEF cells (NIH-3T3) were subcultured in 13 mm confocal dishes one day prior to transfection. On the day of transfection, we transiently transfected cells with 500 ng of SOX2-T2A-mCherry and p300-T2A-GFP plasmids (total 1 μg of plasmids) using TurboFect (Thermo Fisher Scientific, Waltham, MA, USA). After incubating for one day for MEF cells or three days for mESCs, we imaged live cells using a Zeiss LSM 710 confocal microscope (Carl Zeiss, Germany). To study transcriptional activation using the PCP-PP7 system, we transiently transfected cells with 250 ng of each plasmid (SOX2-T2A-mCherry, p300-T2A-GFP, NLS-tdPCP-CFP and pGL3-PP7-SE, total 1 μg of plasmid) on the day of transfection using TurboFect (Thermo Fisher Scientific, Waltham, MA, USA). After one day of incubation, we imaged live cells as described above. mESCs were always cultured in the presence of leukemia inhibitory factor (MERCK, Rahway, NJ, USA). Acquired images were subsequently analyzed using ImageJ software. Pearson's coefficient was calculated using JACoP (Just Another Colocalization Plugin) in ImageJ with default settings.

### Fluorescence recovery after photo-bleaching (FRAP) assay

FRAP assays of SOX2-Cy3 condensates *in vitro* or SOX2-mCherry condensates in live cells were performed using a Zeiss LSM710 confocal microscope (Zeiss, Oberkochen, Germany). Region of interest (ROI) was drawn in condensates and reference region was drawn in other condensates in the nucleus. Background region was drawn in non-condensates in the nucleus. ROI was bleached for 50 iterations at 100% laser power (488 and 561 nm). Images were collected for 5 min with 1 second interval. After background subtraction and correction using intensity from background region and reference region, ROI intensity was normalized to pre-bleach intensity. For curve fitting, at least 10 droplets were used. FRAP recovery curves were fitted to the double exponential function in GraphPad Prism software v.8.3.0 (GraphPad software, Boston, MA, USA).

### Preparation of nucleosomes and chromatins

*Xenopus* histone octamer, ACF complex composed of N-terminal FLAG-tagged *Drosophila* ACF1 and untagged *Drosophila* ISW1, and N-terminal 6хHis-tagged mouse NAP-1 for nucleosome and chromatin assembly were kindly provided by Prof. Jaehoon Kim (Korea Advanced Institute of Science and Technology (KAIST), Daejeon, Korea). Purification of these proteins has been described previously (41). To prepare nucleosomes, 360 ng of SM, dSM or NM was pre-incubated with 400 ng of *Xenopus* histone octamer in 10 mM Tris–HCl (pH 7.5), 2 M NaCl, 2 mM DTT and 2 mM EDTA. After incubation at 4°C for 1 h, the mixture was dialyzed against 10 mM Tris–HCl (pH 7.5) and 20 mM NaCl. Assemblies of chromatins were assisted by an ACF complex (42). Briefly, 360 ng of histone octamer and 2.4 μg of NAP-1 were incubated in 55 μl HKEG buffer (25 mM HEPES–NaOH, pH 7.5, 50 mM KCl, 0.1 mM EDTA and 10% glycerol) for 30 min on ice. After incubation, DNA-ACF mixture (350 ng of plasmid DNA, 70 ng of ACF complex, 23 mM MgCl_2_ and 3.2 mM ATP in 15 μl HKEG buffer) were added and incubated at room temperature for 4 h. Chromatin assembly was confirmed by atomic force microscopy (AFM) using a JPK Nanowizard ULTRA speed instrument (Bruker, Billerica, MA, USA) and EMSA.

### *In vitro* acetylation (IVA) assay

Chromatin assembled with the pGL3 reporter was used in the IVA assay. Chromatin (360 ng) was first incubated with SOX2 (0, 100 and 200 ng) in a binding buffer. After incubation at room temperature for 30 min, 50 ng of p300 (Protein One, Gaithersburg, MD, USA) and 30 μM (final) of acetyl-CoA were added to the mixture and further incubated at 30°C for 30 min. The reaction was stopped by adding 2× SDS sample buffer followed by heating for 5 min at 95°C. Samples were analyzed by western blotting. Experiments were performed at leastthree times.

### Structural modeling and binding simulation

A structure of SOX2_DBD_ was prepared by extracting coordinates from the crystal structure of the SOX2/OCT4/DNA ternary complex (PDB:1GT0). A structural model of human KIX was constructed with SWISS-MODEL using the crystal structure of mouse KIX using CREB-binding protein (PDB:6DMX) as a template (43). Protein-protein docking simulations of DBD_SOX2_ and KIX were performed using ClusPro server (44). The stereochemical quality of results was confirmed using PROCHECK software (45). PyMOL was used for graphical presentation.

### Spheroid formation of OE33 cells, fluorescence-activated cell sorting (FACS) and quantitative real-time (qRT)-PCR analysis

For inducing cancer stem cells (CSCs) from OE33 cells, 1 × 10^6^ cells were inoculated into ultra-low attachment 75 cm^2^ U-flask (CORNING, Corning, NY, USA). After 48 h of incubation, cells were detached using Accutase (StemCell Technologies, Vancouver, BC, Canada) for aldehyde dehydrogenase (ALDH) and qRT-PCR analyses. For ALDH analysis, an ALDEFLUOR kit (StemCell Technologies, Vancouver, BC, Canada) was used with BODIPY-aminoacetaldehyde (BAAA) as a substrate. Cells (1 × 10^6^ cells) were incubated with BAAA at 37°C for 1 h, washed and analyzed using a FACSCanto II (BD Biosciences, San Jose, CA, USA). For the negative control, Diethylaminobenzaldehyde (DEAB), a specific ALDH inhibitor, was co-incubated. For qRT-PCR analysis, total RNA was extracted using an RNeasy Mini Kit (QIAGEN, Hilden, Germany). Total RNA (1 μg of total RNA was reverse transcribed using an RNA-to-cDNA EcoDry Premix (Oligo dT) kit (Takara, Kyoto, Japan). qRT-PCR was performed using iTaq Universal SYBR Green Supermix (Bio-Rad, Hercules, CA, USA). Primers used for qRT-PCR are listed in Supplementary Table S1. Relative RNA amounts were calculated using the 2^–ΔΔCt^ method (46).

For RNA interference to inhibit the expression of SOX2 in OE33 cells, 0.2 × 10^6^ cells were inoculated into 24-well ultra-low attachment plates (CORNING, Corning, NY, USA) with SOX2 siRNA and negative control siRNA (S13294; Invitrogen, Waltham, MA, USA), which were pre-incubated for 20 min in FuGENE reagent (Promega, Madison, WI, USA). After incubation at 37°C for 24 h in a CO_2_ incubator, cells were analyzed.

## Results

### Identification of SOX2-enriched SEs and coactivators using bioinformatic analyses

**Figure 1.**
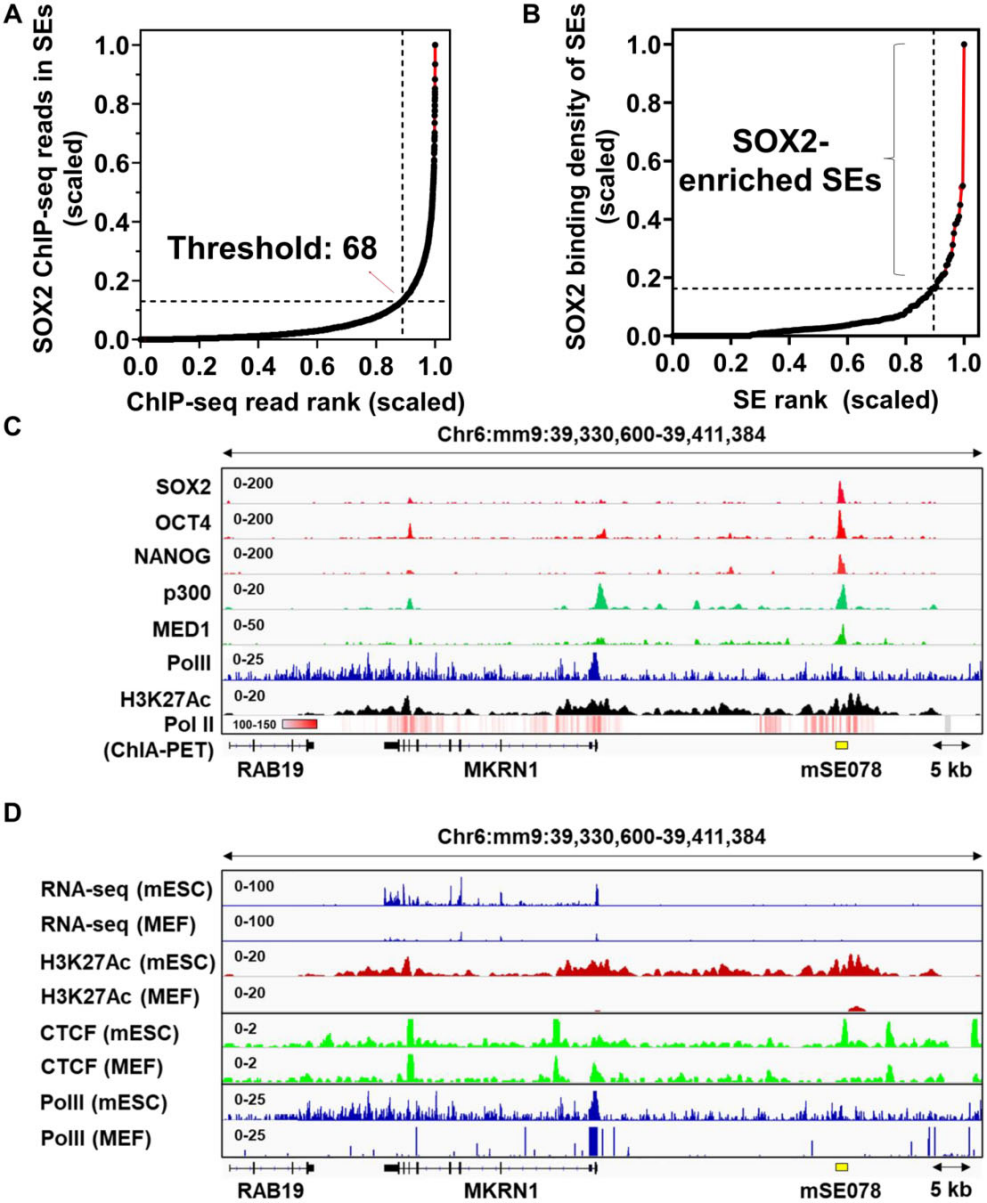
Identification of SOX2-enriched SEs and genomic analysis near *MKRN1* TSS. (**A**) A plot of a total SOX2 chromatin immunoprecipitation followed by sequencing (ChIP-seq) reads (raw reads) in 231 mESCs-SEs vs. a ranked order of reads by increasing value. The x and y axis were scaled from 0 to 1.0. Threshold value ‘68′ was geometrically determined at which the slope of tangent line was 1.0 in the scaled plot. A SOX2 binding density of 231 mESCs-SEs (the counts of SOX2 ChIP-seq reads higher than 68 in SEs/the length of SEs) was calculated. (**B**) A plot of a SOX2 binding density of SEs vs. a ranked order of SEs by a SOX2 binding density. The x and y axis were scaled from 0–1. SEs above a line with a slope of 1 tangent to the scaled plot were identified as SOX2-enriched SEs. (**C**) ChIP-seq profiles (raw reads) of SOX2, OCT4, NANOG, p300, MED1, PolII and H3K27ac and chromatin interaction assay paired-end tag (ChIA-PET) profile (raw reads) of RNA Pol II near *MKRN1* gene in mESCs. mSE078 is indicated by a yellow bar. (**D**) RNA-seq (raw reads) and ChIP-seq profiles (raw reads) of H3K27ac, CTCF and PolII at *MKRN1* gene in mESCs and MEF cells. Integrated genomic viewer (IGV) software was used for preparing images.

To identify SOX2-enriched SEs, we first calculated the SOX2 binding density of previously annotated 231 MED1-SEs in mESCs by dividing counts of SOX2 ChIP-seq reads higher than the read threshold in SEs (GSM1082341) by SE length (kb) (9). The read threshold was geometrically determined at the point of a tangent line with a slope of 1 to the scaled plot of SOX2 ChIP-seq reads in 231 SEs vs. a ranked order by increasing the value (Figure 1A). The read threshold was found to be 68, corresponding to (0.89, 0.13) in the scaled plot (Figure 1A). SOX2 binding density of 231 SEs was then calculated and plotted in ranked order by increasing SOX2 binding density (Figure 1B). We defined SEs located above a tangent line with a slope of 1 to the curve as SOX2-enricehd SEs in the scaled plot. Among 231 SEs, 31 were classified as SOX2-enriched SEs with an average SOX2 binding density of 4.11 (Figure 1B). The remaining 200 SEs had an average SOX2 binding density of 0.41 (range, 0–1.90).

SOX2-enriched SEs are located near genes known to be essential for stemness, neural and retinal development (Supplementary Figure 1), suggesting that SOX2 mediates its known physiological functions by regulating SOX2-enriched SE genes (47–50). Among identified SOX2-enriched SEs, mSE078 was finally chosen as a model SOX2-enriched SE for further experiments because it was the second highest SOX2-enriched SE located in the non-coding region with only one gene (*MKRN1*) within 100 kb distance from the center of SE.

It was found that mSE078, a single constituent SE with a length of 1208 bp, was located 25.8 kb upstream from *MKRN1* transcription start site (TSS) (Figure 1C). Since direct interaction between mSE078 and MKRN1 promoter has been reported in RNA Pol II chromatin interaction assay paired-end tag (ChIA-PET) data (GSM4041604), direct regulation of mSE078 on MKRN1 expression is expected (Figure 1C). Interestingly, unlike in mESCs, RNA-seq and H3K27ac peaks near *MKRN1* in MEF cells were not observed, suggesting that mSE078 could regulate *MKRN1* in a cell type-specific manner (Figure 1D). To date, many coactivators and mediators have been reported to work together with pioneer TFs in SEs (18,51–53). Our genomic analysis using publicly available ChIP-seq data of mESCs revealed that the occurrence of SOX2 and p300 was highly correlated with each other at mSE078 (Figure 1C). Consistently, p300 was identified in the SOX2 interactome. SOX2 is known to be a substrate for p300 (54). Therefore, it is conceivable that SOX2 can recruit p300 and organize transcriptional activation with p300 at SOX2-enriched SEs for SE-mediated gene regulation. Co-localization of SOX2 and p300 was also observed in other SOX2-enriched SEs such as mSE219 and mSE191 (Supplementary Figure 2A). However, we found that their co-localization was relatively weak in non-SOX2-enricehd SEs (Supplementary Figure 2B).

To validate the involvement of SOX2 in recruiting p300 and its effect on enhancer activity within SOX2-enriched SEs, we performed chromatin immunoprecipitation followed by quantitative PCR (ChIP-qPCR) analysis. Specifically, we analyzed three SOX2-enriched SEs (mSE078, mSE191 and mSE219) and two non-SOX2-enriched SEs (mSE224 and mSE227) in mESCs with SOX2 knockdown (Supplementary Figure 3A). First, we confirmed a higher SOX2 occupancy in SOX2-enriched SEs than in non-SOX2-enriched SEs, while the binding of p300 and RNA Pol II and the level of H3K27ac were similar among all SEs. Upon SOX2 knockdown, occupancies of both SOX2 and p300 were observed to decrease across all SEs in mESCs (Supplementary Figure 3A, B). Notably, reductions were more pronounced in SOX2-enriched SEs than in non-SOX2-enriched SEs following SOX2 knockdown in mESCs. Additionally, losses of H3K27ac and RNA Pol II known to be indicative of active transcription were more prominent in SOX2-enriched SEs than in non-SOX2-enriched SEs (Supplementary Figure 3C, D). These findings highlight critical roles of SOX2 in gene regulation through SOX2-enriched SEs, particularly in conjunction with p300. We further conducted subsequent investigations on roles of both SOX2 and p300 in transcriptional condensation and activation using mSE078 as a representative model SE.

### Binding of SOX2 to SOX binding motif of mSE078

**Figure 2.**
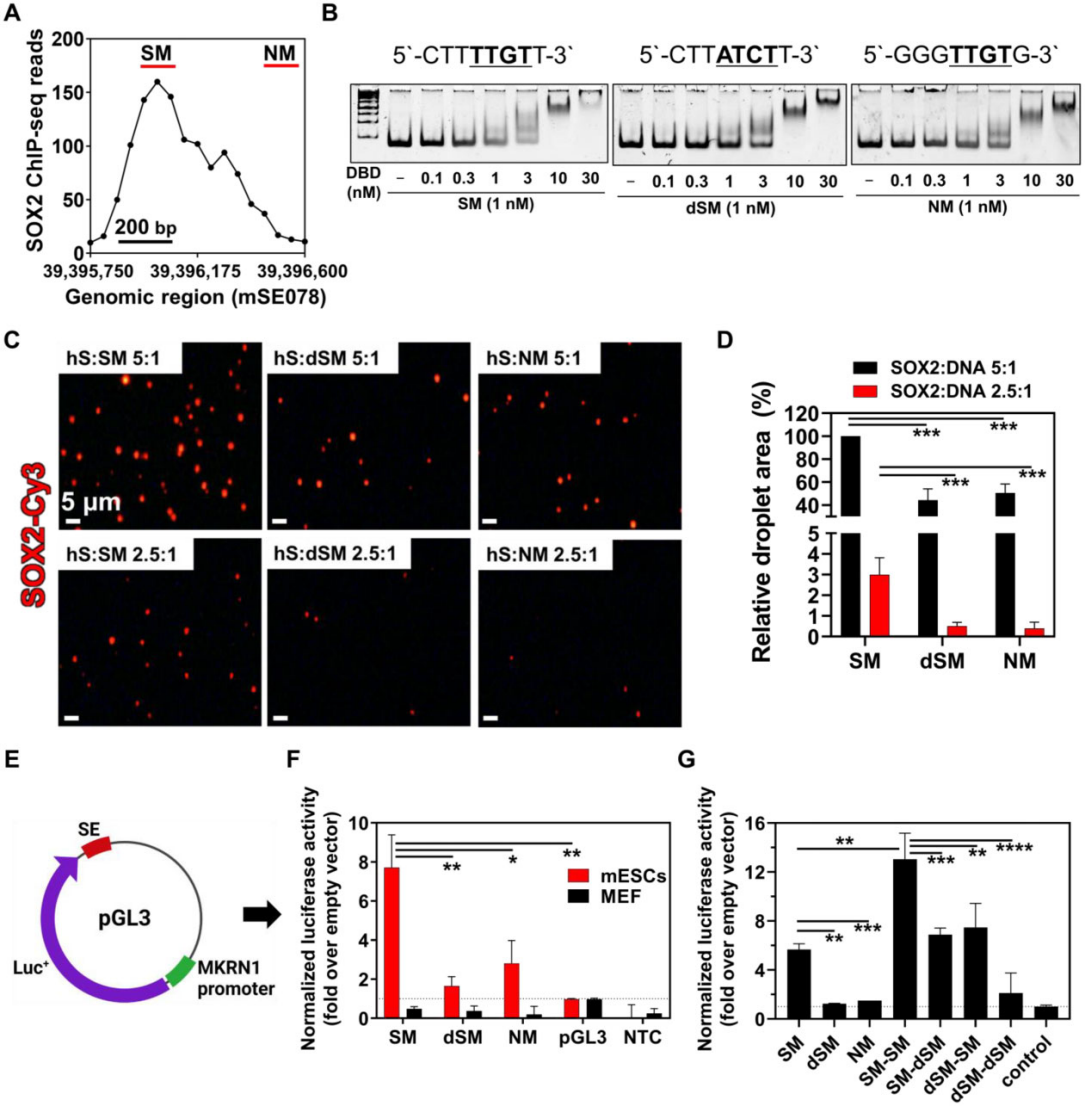
Enhancer activities of mSE078 fragments. (**A**) SOX2 ChIP-seq profile in mSE078. Locations of SM and NM showing the highest and lowest SOX2 ChIP-seq reads, respectively, are underlined with red bars. (**B**) Electrophoretic mobility shift assay (EMSA) of SOX2_DBD_/DNA complexes. Sequences of each tested SOX2 biding motif in SM, dSM and NM are shown above gel images. Various concentrations of SOX2_DBD_ were tested for 1 nM DNA motifs. (**C**) Comparison of SM, dSM and NM in DNA-dependent SOX2 condensation. 837 nM (5:1) or 419 nM (2.5:1) SOX2-Cy3 and 162 nM DNA were incubated in PS buffer. After 1 h of incubation, images were analyzed. (**D**) Total area of droplets was quantified as described in materials and methods. Values are relatively shown as the maximum value (SOX2:SM 5:1). (**E**) A schematic description of a luciferase reporter plasmid containing *MKRN1* promoter and SM, dSM or NM. (**F**) Luciferase activities of pGL3-SM, dSM and NM in mESCs and MEF cells. (**G**) Luciferase activities of pGL3 reporter plasmids containing differently combined SM and dSM in mESCs. All luciferase activities were normalized to Renilla luciferase activity from co-transfected pRL-TK plasmid. Their relative values to the activity of empty pGL3 reporter plasmid are shown. For all experiments, error bars indicate standard errors of means (SEM) of three independent experiments. *P*-values were calculated using a two-tailed *t*-test. (ns) *P* < 0.1234; (*) *P*< 0.0332; (**) *P* < 0.0021; (***) *P*< 0.0002; (****) *P*< 0.0001.

Condensation of SOX2 with DNA has been reported using bacteriophage λ DNA as a model DNA (35). Considering local enrichment of SOX2 in SOX2-enriched SEs, it was hypothesized that SOX2 could form condensates with DNA sequences in SOX2-enriched SEs. To test this hypothesis, we assessed the formation of SOX2 condensates with DNA using DNA fragments from mSE078 *in vitro*. As SOX2 binding density was not identical across mSE078, we selected two DNA fragments, SOX2 motif (SM) and Non-SOX2 motif (NM), that showed the highest and lowest binding densities, respectively, for comparative experiments (Figure 2A). The length of SM and NM was set to 172-bp for a mono-nucleosome assembly experiment (55). In SM, there was only one consensus SOX2 binding motif (5′-CTTTTGTT-3′). Notably, NM also had one consensus SOX2 binding motif (5′-GGGTTGTG-3′), although it showed a lower SOX2 binding density. As a control, the consensus motif of SM was substituted with 5′-CTTATCTT-3′ to generate dSM. We tested binding affinities of the DNA-binding domain of SOX2 (SOX2_DBD_; aa 39–127) to SM, dSM and NM using an electrophoretic mobility shift assay (EMSA). EMSA results revealed that SOX2_DBD_ showed a reasonable binding affinity to SM, but not to dSM (Figure 2B and Supplementary Figure 4A), suggesting that the SOX2 binding motif was essential for the binding of SOX2 to mSE078. In addition, the binding of SOX2_DBD_ to NM was significantly weaker than that to SM, despite the presence of a consensus SOX2 binding motif. While 71% of SM was occupied by SOX2 in a SOX2_DBD_:DNA ratio of 3:1, only 38% of NM was bound to SOX2_DBD_ under the same conditions (Figure 2B and Supplementary Figure 4A). Given that the difference between the SOX2 binding motif in SM (5′-CTTTTGT-3′) and NM (5′-GGGTTGT-3′) occurs at 5′-flanking region of SOX2 binding motif, our results are consistent with a previous study reporting that SOX2 binding motifs containing purine–purine base stacking at 5′-flanking region are unsuitable for SOX2 binding, which can lead to a shorter SOX2 residence time (56). The consistency of our results with these previous observations was further supported by analysis of SOX2 binding density for each SOX2 binding motif (Figure 2A).

### Condensation of SOX2 with SOX2 binding motif of mSE078

As the binding of SOX2 to SM was confirmed, we next investigated SOX2 condensation with SM. In previous studies, SOX2 condensation has been demonstrated in physiologically irreverent conditions due to a high SOX2 concentration (10–40 μM) and too many repeats of the SOX2 binding motif in the model DNA used for condensation (24,35). To investigate the formation of SOX2 condensates in cells, we ectopically expressed mCherry-labeled SOX2 in mESCs and confirmed the presence of SOX2 condensates (Supplementary Figure 4B). The liquid nature of SOX2 condensates was verified by observing a rapid recovery of mCherry signals after photobleaching (Tau = 10 s) (Supplementary Figure 4C, D). Time-course analysis of droplets revealed fusion of small droplets in mESCs (Supplementary Figure 4E). We then demonstrated condensation of SOX2 with mSE078 *in vitro*. It is known that cellular concentration of SOX2 in the nucleus is approximately 1–1.3 μM (57) and that only a limited number of SOX2 binding motifs are present near SOX2 peaks in SE shown by ChIP-seq analysis (9,58). Thus, we applied them as physiologically relevant conditions for testing SOX2 condensation with DNA. We prepared Cy3-labelled SOX2 (SOX2-Cy3) in a buffer containing 10% polyethylene glycol (PEG) 8000, the most commonly used crowding reagent, to monitor SOX2 condensation based on formation of phase-separated droplets under conditions mimicking the cellular environment as reported in previous studies (59–61). When SOX2-Cy3 was incubated alone at a near physiological concentration (0.84 μM) in the presence of the crowding reagent, no foci were observed (Supplementary Figure 4F). Under the same conditions, no foci were observed when the SOX2-Cy3 concentration was increased to 13 μM (data not shown). However, incubation of 0.84 μM SOX2-Cy3 with SM resulted in the formation of phase-separated droplets that were visible by bright-field and fluorescence microscopy (Supplementary Figure 4F), suggesting the formation of SOX2 condensates. Foci were detected even when unlabeled SOX2 combined with 5% of SOX2-Cy3 (total 0.84 μM) was incubated with SM, indicating that condensation was not caused by the fluorescence dye itself (Supplementary Figure 4G). In previous studies, phase-separated droplets were considered as liquid-like condensates when they showed distinctive biophysical properties such as fluorescence recovery after photobleaching (FRAP), droplet fusion and dissolution by 1,6-hexanediol, a hydrophobic interaction inhibitor (24,62,63). Accordingly, in the current study, the high intensity foci or phase-separated droplets observed under *in vitro* conditions were considered as liquid-like condensates since they showed the following properties: (1) high-intensity foci displayed liquid-like property as demonstrated by FRAP assay (Tau = 42.5 s) (Supplementary Figure 4H, I); (2) droplet fusion occurred (Supplementary Figure 4J); and (3) high-intensity foci were effectively dissolved when treated with 1,6-hexanediol (Supplementary Figure 4K, L).

We further revealed that physiological salt concentrations (75–150 mM) were required for SOX2 condensation with DNA (Supplementary Figure 4M–P). Therefore, the following conditions were used for condensation experiments unless otherwise stated: 20 mM Tris–HCl (pH 7.5), 80 mM NaCl, 5 mM MgCl_2_ and 10% PEG8,000. We further observed that SOX2 condensation with DNA occurred in a certain range of DNA and salt concentration (Supplementary Figure 4Q, R). Next, we tested condensation of SOX2 using dSM and NM at different ratios. Although SOX2 formed condensates with dSM or NM as observed by the formation of fluorescence foci, they were less significant than those with SM (Figure 2C, D). These results suggest that SOX2 can form condensates with DNA in an affinity- and ratio-dependent manner, although non-specific binding of SOX2 to DNA can also affect the condensation process.

### SOX2- and SOX2 binding motif-dependent enhancer activity of mSE078

To link the condensation property of SOX2 with SM to enhancer activity of mSE078, we performed a luciferase reporter assay using pGL3 luciferase reporter plasmids containing DNA fragments from mSE078 and *MKRN1* promoter in both mESCs and MEF cells (Figure 2F, G). In mESCs, the SM-containing reporter showed the highest enhancer activity, whereas dSM- and NM-containing reporters exhibited much lower activities (Figure 2F), consistent with their ability to form SOX2 condensates with them (Figure 2C, D). To further investigate the motif-dependency of enhancer activity, we created a new DNA fragment, NM(SM), in which a SOX2 binding motif in NM (5′-GGGTTGTG-3′) was replaced with that in SM (5′-CTTTTGTG-3′). We observed that the NM(SM)-containing reporter generated *de novo* enhancer activity in mESCs, demonstrating that enhancer activities of these DNA fragments were profoundly associated with their binding affinity to SOX2 (Supplementary Figure 5A). To further investigate the exclusive role of SOX2 binding motif in enhancer activity, we constructed artificial enhancers by combining SM and dSM in various orders in reporter plasmids and checked the effect of duplication of TF motifs on transcription (Figure 2G). The reporter with additional SM in the enhancer exhibited increased enhancer activity. However, additional dSM showed no deteriorating effect on the activity. These results demonstrate that enhancer activity of mSE078 is highly associated with the number of SOX2 binding motifs, which have a high affinity for SOX2.

While the reporter activity was highly dependent on the species of enhancer sequence in mESCs, no enhancer activity was observed in MEF cells possibly due to the lack of SOX2 expression in MEF cells (Figure 2F). To test this hypothesis, we examined enhancer activity after ectopic expression of SOX2 in MEF cells. Similarly, we tested effects of other pioneer TFs on reporter expression by expressing OCT4 and NANOG as controls. Interestingly, a sequence-dependent enhancer activity was observed in MEF cells in the presence of SOX2 expression (Supplementary Figure 5B). However, enhancer activity was not observed when OCT4 or NANOG was expressed (Supplementary Figure 5C). In addition, co-expression of OCT4 and/or NANOG with SOX2 did not show any synergistic effect on enhancer activity (Supplementary Figure 5D). These results validate the exclusive role of SOX2 in transcriptional activation of mSE078, similar to the OCT4-specific vulnerability of OCT4-enriched enhancers (64). Current results also suggest that enhancer activities of SEs could be dependent on certain pioneer TFs that are highly enriched in SEs. Considering the roles of SM in both SOX2 condensation and enhancer activity, it is conceivable that SOX2-dependent enhancer activity of mSE078 is highly correlated with SOX2 condensation on mSE078.

### Evaluation of p300 interaction with SOX2

**Figure 3.**
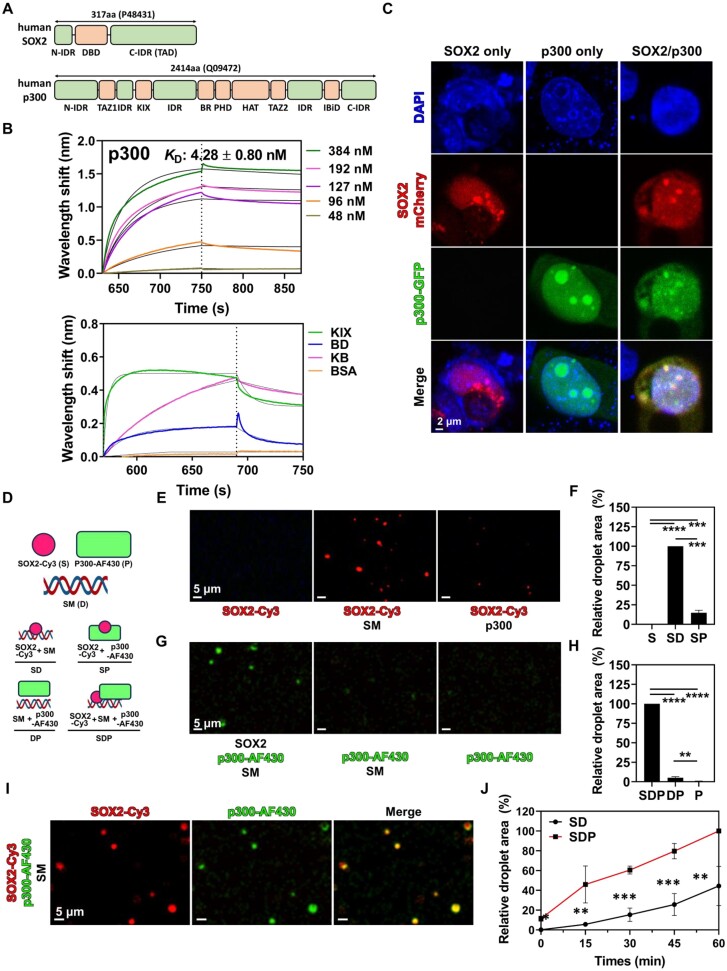
Bio-layer interferometric (BLI) analysis and co-condensation of SOX2 and p300. (**A**) Schematic representation of domains of human SOX2 (Uniprot ID: P48431) and human p300 (Uniprot ID: Q09472). IDR: intrinsically disordered region. (**B**) Representative bio-layer interferometric (BLI) curves of the interaction between SOX2 and p300 (top) and the interaction between SOX2 and p300 fragments (bottom). Dissociation constant (*K*_D_) was determined by globally fitting the curve to the 1:1 binding model. Concentrations of p300 fragments used in the assay are as follows: 3 μM KIX and BR; 0.8 μM KB and 6 μM BSA. All experiments were performed in triplicate. (**C**) Confocal microscopic analysis of mESCs expressing either SOX2-mCherry, p300-GFP or both. (**D**) Schematic representation of the *in vitro* condensation assay. SOX2-Cy3 (S), p300-AF430 (P) and SM (D). S: SOX2 alone; P: p300 alone; SD: SOX2:DNA; SP:SOX2:p300; SDP: SOX2:DNA:p300. (E–H) Fluorescence (FS) microscopic analysis of (**E**) SOX2 and (**G**) p300 condensation and (F, H) total area of droplets. SOX2-Cy3 (837 nM), SM (126 nM) and p300-AF430 (126 nM) were used. The total area of droplets was quantified as described in materials and methods. Relative values to values of SD (**F**) and SDP (**H**) are shown. (**I**) FS analysis of co-condensation of SOX2 and p300 in SOX2:SM condensates. SOX2-Cy3 (558 nM), SM (100 nM) and p300-AF430 (126 nM) were co-incubated in PS buffer. (**J**) Time-course analysis of SOX2:DNA and SOX2:DNA:p300 droplets. Samples were collected every 15 min and imaged. The total area of droplets was quantified as described in materials and methods. Counts are relatively shown as the maximum value (SD, 60 min). For all experiments, particles were analyzed from five randomly chosen regions. Error bars indicate standard errors of means (SEM) of at least three independent experiments. *P*-values were calculated using *t*-test. (ns) *P* < 0.1234; (*) *P*< 0.0332; (**) *P* < 0.0021; (***) *P*< 0.0002; (****) *P*< 0.0001.

As p300 was co-localized with SOX2 in mSE078 (Figure 1C), we anticipated that p300 would cooperatively contribute to SOX2-mediated transcriptional condensation and activation of mSE078. We first investigated direct interaction of SOX2 with p300 using a bio-layer interferometric (BLI) analysis and established a strong association between SOX2 and p300 with *K*_D_ of 4.28 nM (Figure 3B, top), which was almost comparable to reported *K*_D_ values for SOX2/dsDNA and SOX2/nucleosomes interactions of 1.46–2.1 and 0.34–1.43 nM, respectively (58,65). Various domains, including KIX, bromodomain (BD) and histone acetyltransferase (HAT) domains, are present in p300 (Figure 3A). Since the KIX domain is known to play a crucial role in binding to various TFs such as c-MYB, TAX and NANOG (3,66,67), we investigated the interaction between SOX2 and KIX domain by pull-down assay using biotinylated SM. For comparative analysis, we used p300 variants including p300_KIX_ (residues 566–646), p300_KB_ (KIX + BD, residues 566–1139) and p300_BH_ (BD + HAT, residues 1067–1663) (Supplementary Figure 6A). From these analyses, we confirmed that KIX was the main interacting domain for SOX2 (Supplementary Figure 6B).

To further explore the role of p300 domains in SOX2 binding, we analyzed the binding between SOX2 and p300 domains using BLI (Figure 3B, bottom). We found that p300_BD_ exhibited a 20.1-fold higher *K*_D_ of SOX2 than p300_KIX_ (Figure 3B and Supplementary Table 2). In contrast, p300_KB_ showed 41.4- and 834-fold lower *K*_D_ to SOX2 than p300_KIX_ and p300_BD_, respectively. These BLI results suggest that each domain of p300 has a distinct binding affinity for SOX2. This is highly reminiscent of the p300:p53 interaction in which p53 tetramer interacts with various domains of p300 with distinct binding affinities (68,69). To gain insights into the binding mode of SOX2 to p300, associations of SOX2_DBD_ and the C-terminal domain of SOX2 (SOX2_CTD_) containing a transactivation domain (TAD) with p300_KIX_ or p300_BD_ were assessed (Supplementary Figure 6C). BLI results showed that SOX2_DBD_ was strongly associated with p300_KIX_ but weakly associated with p300_BD_ (Supplementary Table 3). In contrast, SOX2_CTD_ showed much weaker binding to p300_KIX_ with almost no binding to p300_BD_, suggesting that SOX2_CTD_ could mediate weak and transient interactions with p300 domains probably through TAD, similar to many other p300-associating TFs (68,70,71). These results demonstrate that SOX2_DBD_ and p300_KIX_ play key roles in the direct interaction between SOX2 and p300. However, it is noteworthy that SOX2_CTD_ seems to mediate weak interactions with p300, which contributes to multivalent protein–protein interactions important for co-condensation.

To demonstrate the formation of co-condensates of SOX2 and p300, we ectopically expressed mCherry-labeled SOX2 and GFP-labeled p300 either alone or both in various cells. Results confirmed that SOX2-mCherry formed condensates in the nucleus of mESCs (Figure 3C, SOX2 only, p300 only). However, in MEF cells, SOX2-mCherry showed a diffused pattern, although it formed some concentrated regions or foci (Supplementary Figure 6D, SOX2 only). It has been consistently reported that condensates are not well formed when SOX2 is expressed in HEK 293T cells and MEF cells (28,72). Interestingly, SOX2 localization in both nuclear and cytoplasm in MEF cells was observed, which is similar to the immunocytochemistry results from the previous studies (54,73). These results suggest the possible role of SOX2 other than a transcription factor, as reported in other studies (65,74). In the case of p300-GFP expression in cells, we found that p300-GFP mostly existed as condensates in MEF cells (Supplementary Figure 6D, p300 only). This result was consistent with the condensation pattern of p300 in mESCs, U2O2 cells and HeLa cells (34,75). When SOX2-mCherry and p300-GFP were co-expressed, co-localization of SOX2 condensates with some p300 condensates were observed in mESCs (Figure 3C, SOX2/p300). However, when they were co-expressed in MEF cells, although they were co-localized as judged by the overlap between the GFP and mCherry signal profile, the size and the shape of condensates were different from those in MEF cells expressing p300-GFP alone (Supplementary Figure 6E, SOX2/p300, yellow arrow). Overall, these results suggest that p300 distribution is highly dependent on SOX2 in cells due to a direct interaction of p300 with SOX2.

### Role of p300 in DNA-dependent SOX2 condensation

We further analyzed interactions of p300 and its domains with SOX2 and nucleosomes reconstituted with SM using EMSA. Results revealed that p300 showed supershifts of the SOX2:nucleosome complex, although it did not display a direct interaction with nucleosomes (Supplementary Figure 6F). In comparison with p300_KB_ and full-length p300, p300_KIX_ alone was unable to induce a supershift of the nucleosome at a molar ratio of 1:2 (SOX2:p300_KIX_). This observation could be attributed to the lower binding affinity of p300_KIX_ to SOX2 as shown in Supplementary Table 2. Indeed, we were able to confirm that p300_KIX_ could induce a supershift of nucleosome when a higher amount of p300_KIX_ (at a ratio of 10:1 to 30:1 p300_KIX_:SOX2) was used (Supplementary Figure 6F). As p300 did not bind to free DNA or nucleosomes, under the current experimental conditions, SOX2 seemed to recruit p300 to SOX2-bound DNA and nucleosomes through a direct interaction with p300. It was, therefore, notable to determine the role of p300 in SOX2 condensation with DNA. To test *in vitro* co-condensation of SOX2 and p300, we used Cy3-labeled SOX2 (red) and AF430-labeled p300 (green). We found that p300 alone had a minimal effect on the formation of SOX2 condensates in the absence of DNA (Figure 3D, E). In addition, it was also observed that p300 alone could not form condensates with DNA in the absence of SOX2 (Figure 3F, G). Further time-course analysis of SOX2 condensation with DNA in the presence or absence of p300 revealed that p300 significantly influenced condensation kinetics by accelerating the formation of SOX2 condensates (Figure 3I). p300 also increased fluorescence intensity, number and sizes of droplet (Supplementary Figure 7A–C). Furthermore, p300 improved the recovery rate of Cy3 signals (SOX2) in condensates during FRAP analysis (Supplementary Figure 7D). Collectively, our results provide compelling evidence for direct recruitment of p300 to SOX2:DNA or SOX2:nucleosome complex by SOX2, which enhances SOX2 condensation with DNA and increases liquid property of SOX2 condensates.

### Recruitment of p300 to chromatin by SOX2 for transcriptional activation of mSE078

**Figure 4.**
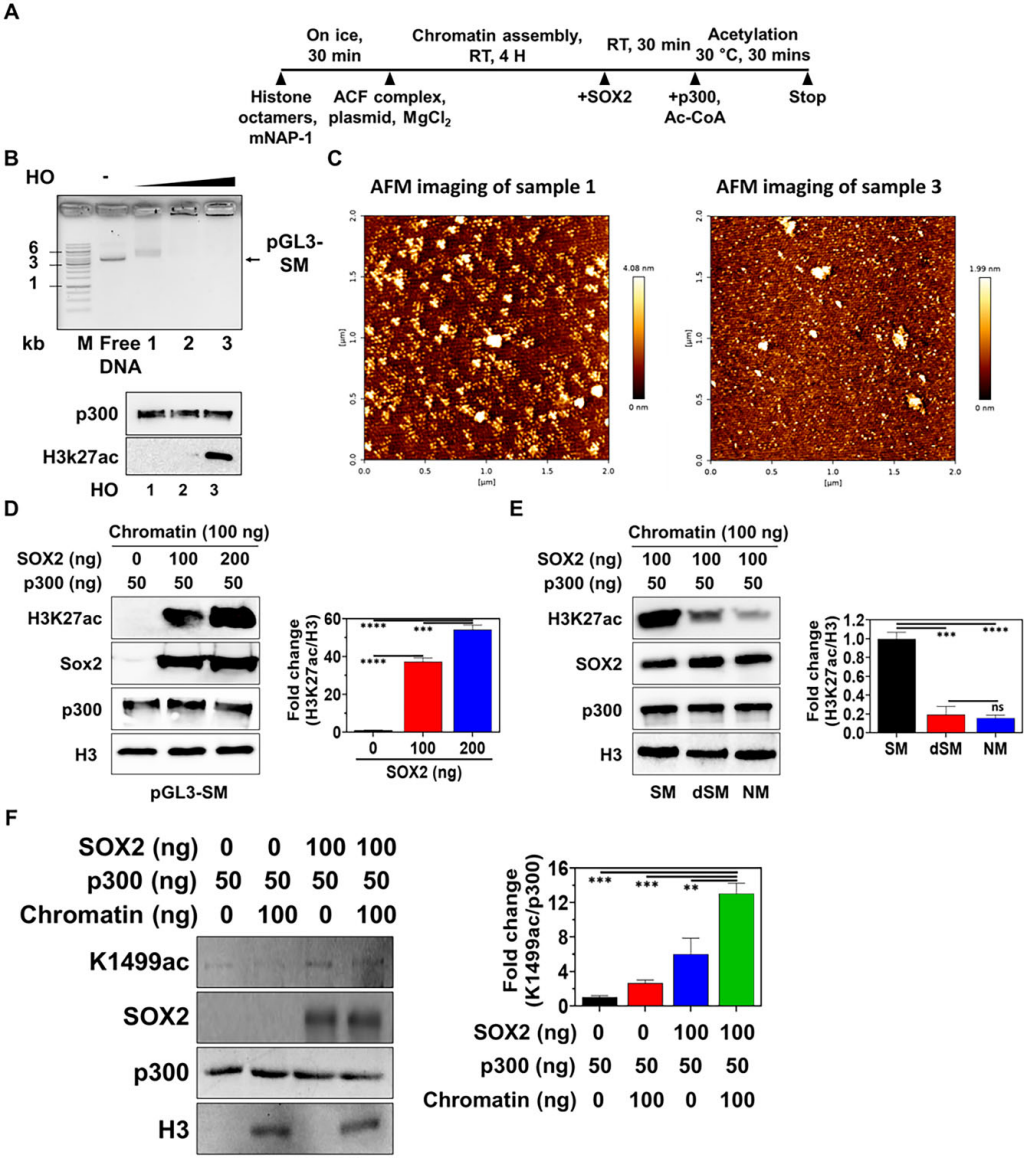
*In vitro* chromatin acetylation (IVA) and p300-autoacetylation. (**A**) An experimental scheme of IVA assay. (**B**) Chromatin assembly with different HO amounts. Top: electrophoretic mobility shift assay (EMSA) of chromatin assembly of pGL3-SM; Bottom: IVA of chromatin assembly of pGL3-SM; M: Size marker; HO: histone octamer. In chromatins 1, 2 and 3, the same amount of DNA (360 ng) was mixed with increasing amounts of HO (300, 600 and 900 ng of HO from chromatin 1 to 3, respectively) for chromatin assembly. (**C**) Atomic force microscopic analysis of supernatants of chromatin assembly from samples 1 and 3 of (B, top). (**D**) Left: western blot analysis of p300-mediated H3K27 acetylation (H3K27ac) in chromatin of pGL3-SM with different amounts SOX2, right: H3K27ac intensity (fold change to 0 ng SOX2 result) normalized by H3 intensity. (**E**) Left: western blot analysis of p300-mediated H3K27ac in chromatin of pGL3-SM, dSM or NM, right: H3K27ac intensity (fold change to SM result) normalized by H3 intensity. (**F**) Left: western blot analysis of p300 trans-autoacetylation using anti-Lys1499 acetylation (K1499ac) antibody, right: K1499ac intensity (fold change to no SOX2 and chromatin result) normalized by p300 intensity. p300 acetylation was performed in the presence or absence of SOX2 and chromatin of pGL3-SM. Error bars indicate standard errors of means (SEM) of three independent experiments. *P*-values were calculated using a two-tailed *t*-test. (ns) *P* < 0.1234; (*) *P* < 0.0332; (**) *P* < 0.0021; (***) *P* < 0.0002; (****) *P* < 0.0001.

p300, a HAT, functions as a chromatin remodeler that can activate transcription by acetylating histone tails in nucleosomes. To understand implications of p300 recruitment to SOX2 condensates with DNA in transcriptional activation of mSE078, we investigated p300-mediated chromatin acetylation (H3K27ac) using chromatin assembled with pGL3-SM, -dSM, and -NM using purified SOX2, p300, NAP1 and ACF1 complex (Figure 4A, Supplementary Figure 8). Chromatin assemblies were checked by EMSA and atomic force microscopy (Figure 4B, C). Histone octamers (HO) were mixed with DNA at different ratio to determine the optimal plasmid-to-HO ratio for the chromatin assembly (Figure 4B, top). Chromatin acetylation (H3K27ac) was not observed in chromatin samples 1 and 2, while acetylation was noticed in chromatin 3 (Figure 4B, bottom). Since chromatin 3 was assembled with the excess amount of HO, we interpreted this result as indicating the acetylation of the excessive free HO by p300. Accordingly, the absence of acetylation at chromatins 1 and 2 suggests that chromatin assembled under the current reaction conditions cannot be acetylated by p300 alone. (Figure 4B). However, we observed that acetylation levels on pGL3-SM chromatin increased in a SOX2 concentration-dependent manner (Figure 4D). Acetylation levels were significantly lower in chromatins of pGL3-dSM and -NM than those in chromatin of pGL3-SM (Figure 4E).

It is also known that p300-interacting TFs can facilitate *trans*-autoacetylations of p300, which can activate the catalytic activity of p300 (76,77). Thus, we assessed whether SOX2 could enhance *trans*-autoacetylation of p300 by monitoring acetylation of Lys1499 (K1499-ac) in p300 (77). Consistent with a previous report (78), the level of K1499-ac in recombinant p300 expressed in insect cells was low (Figure 4F). However, when co-incubated with SOX2, the level of p300 autoacetylation was increased (Figure 4F). Current EMSA results showed that SOX2 recruited p300 into SOX2-bound nucleosomes (Figure 4E). These results verify that SOX2 plays multiple roles in transcriptional activation by recruiting p300 into closed chromatin and mediating *trans*-autoacetylation of p300, which might enhance the catalytic activity of p300. Considering weak acetylation levels in chromatins of dSM and -NM compared to those in chromatins of SM (Figure 4E), the cooperation of SOX2 with p300 might be a key mechanism for transcriptional activation of mSE078.

### Validation of p300 and SOX2 interaction

To further validate functional implications of the SOX2:p300 interaction in transcriptional activation, we constructed a docking model of SOX2_DBD_:p300_KIX_ using the ClusPro server with crystal structure of human SOX2_DBD_ (PDB:1GT0) and the p300_KIX_ domain (aa 566–646) of human p300 AlphaFold 2 model (AF-Q09472-F1-model_v.2) (Supplementary Figure 9A) (44). The quality of the energy-minimized model was validated using a Ramachandran plot. The most favored, allowed and disallowed regions were 83.8%, 16.2% and 0%, respectively (79). Notably, when the docking model overlapped with the Cryo-EM structure of the SOX2_DBD_:nucleosome complex (PDB:6T7B) (19), it was found that the KIX binding region of SOX2_DBD_ was opposite to nucleosome binding sites, providing a structural basis for the binding of SOX2 to both nucleosomes and p300 at the same time (Supplementary Figure 9B). The model revealed that SOX2_DBD_ and p300_KIX_ formed a complex via charge-charge and hydrogen bonds, such as Arg53:Glu643, Arg57:Asn607, Glu78:Arg603, Glu86:Arg603/Arg604 and Arg96:Thr594 (Supplementary Figure 9A). Substitution of key charged residues such as Glu78 and Glu86 in SOX2_DBD_ with Ala resulted in reduced binding affinity to p300_KIX_ (Supplementary Figure 9C). Consistently, SOX2 mutants lacking affinity for p300_KIX_ failed to induce p300-dependent H3K27ac signals compared to wild-type SOX2, while maintaining comparable DNA binding activities to wild-type (Supplementary Figure 9D, E). Subsequently, luciferase activity of pGL3-SM with expression of SOX2 mutants was 30% of that with wild-type SOX2 (Supplementary Figure 9F). In contrast, SOX2 mutants containing Ala substitution on control residues (Lys73 and Arg74, respectively) that did not affect binding to p300_KIX_ showed no effect on the enhancer activity of SM (Supplementary Figure 9G, H). Collectively, our results confirmed that direct interaction between SOX2 and p300 was necessary for p300-dependent acetylation of H3K27, a key mechanism of SOX2-dependent chromatin remodeling and subsequent transcriptional activation of mSE078.

### Co-condensation of SOX2 and p300 for transcriptional activation of mSE078

**Figure 5.**
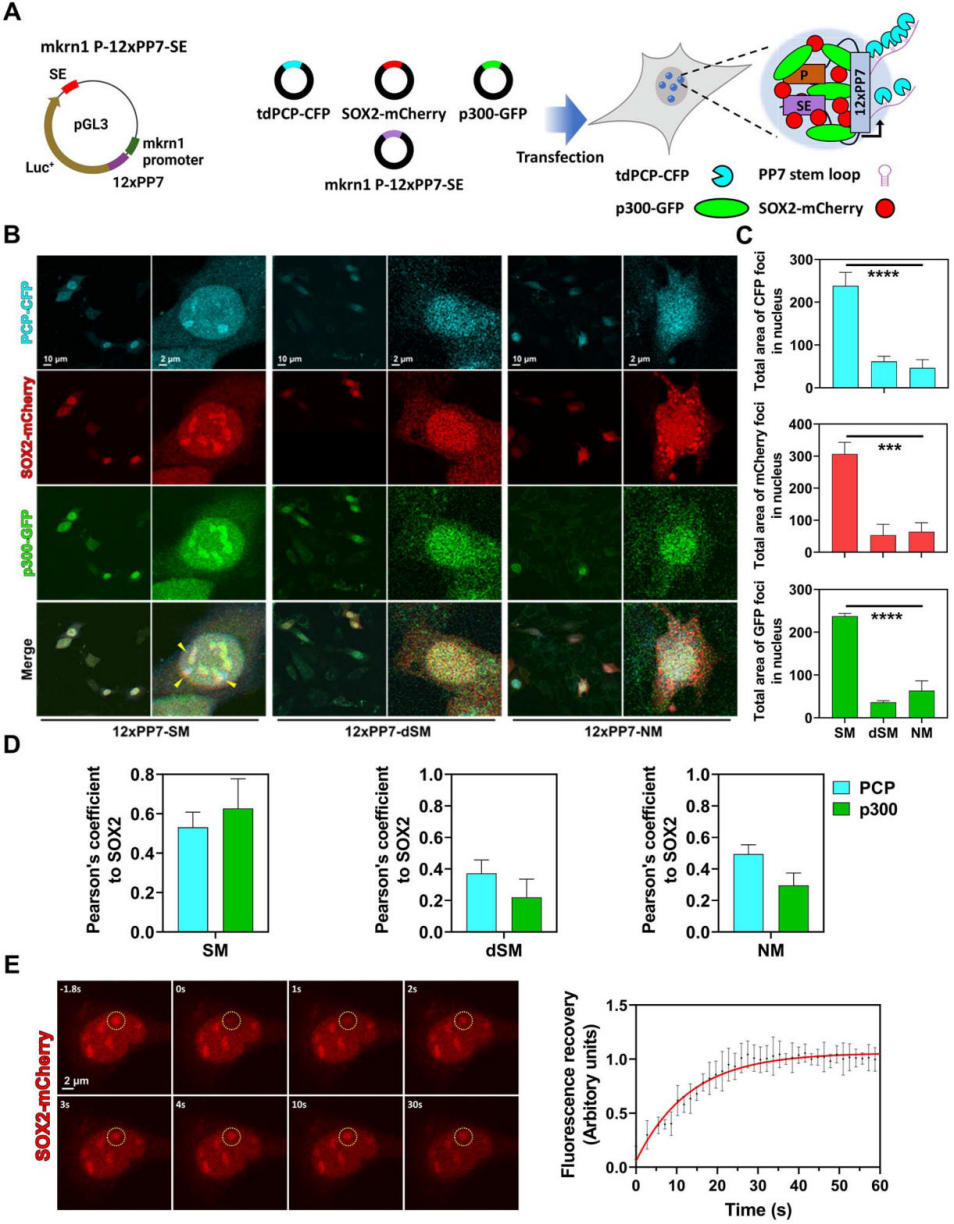
Confocal microscopic analysis of enhancer activity of mSE078 fragments using PCP-PP7 system in MEF cells. (**A**) Left: Schematic representation of mkrn1 *P*-12 × PP7-SE reporter plasmid construct. Right: Schematic representation of PCP-PP7 system used in this study. (**B**) Confocal microscopic analysis of tdPCP-CFP (transcript), SOX2-mCherry and p300-GFP signals in MEF cells. Cells were imaged after 24 h-post transfections of four plasmids. Left: 40× magnification images; Right: closed-up images of single cells. (**C**) Quantification of CFP (PCP), mCherry (SOX2) and GFP (p300) foci. Error bars indicate standard errors of means (SEM) from at least 15 nuclei. Quantification was done using ImageJ software. Particle size larger than 2 × 2 μm^2^ was calculated. (**D**) Pearson's coefficients of CFP and GFP signals to mCherry signals. Pearson's coefficients were calculated using ImageJ software. Error bars indicate SEMs from ten nuclei. (**E**) Fluorescence recovery after photobleaching (FRAP) measuring SOX2-mCherry condensates in MEF cells co-transfected with PCP-CFP, SOX2-mCherry and p300-GFP plasmids. Photobleaching was initiated at 0 s. Curve shows mean (red dot) and SEM (black bar) of mCherry intensity of five regions. FRAP recovery curves were fitted to the double exponential function. *P*-values were calculated using one-way ANOVA. (ns) *P* < 0.1234; (*) *P*< 0.0332; (**) *P* < 0.0021; (***) *P*< 0.0002; (****) *P*< 0.0001.

It has been reported that co-condensation of p300 and transcription factor drives strong transcriptional activation (34,80). To investigate effects of co-condensation of SOX2 and p300 on enhancer activity of mSE078 in cells, we performed co-transfection experiments using gene encoding CFP-labeled PP7 bacteriophage-coat protein (PCP), mCherry-labeled SOX2, GFP-labeled p300 and PP7 reporter plasmids. The PP7 reporter plasmids consist of 172-bp super-enhancer elements from mSE078 and 12 repeats of oligonucleotide (476 bp) encoding PP7 stem-loop RNA (12 × PP7) inserted in the 3′ end and 5′ end of luciferase gene, respectively (Figure 5A, left). The transcript of the 12 × PP7 sequence forms stem-loop structures that could be recognized by PCP-CFP, which results in the formation of CFP foci. Therefore, PP7 reporter plasmid could serve as a transcription reporter (Figure 5A, right). To avoid the effect of endogenous SOX2 on reporter expression, we performed experiments using MEF cells whose endogenous SOX2 expression was lacking or significantly low (Figure 2F). SOX2-mCherry formed condensates only when SM-containing 12 × PP7 reporter plasmid was co-transfected (Supplementary Figure 10A, SOX2-mCherry only). This was also confirmed by differential interference contrast (DIC) imaging (Supplementary Figure 10B). However, cellular localization of PCP-CFP or p300-GFP was not affected by co-transfection with the SM-containing 12 × PP7 reporter plasmid (Supplementary Figure 10A, PCP-CFP only and p300-GFP only). When we co-transfected plasmids encoding PCP-CFP, p300-GFP and SOX-mCherry, respectively, we observed co-localization of SOX2-mCherry and p300-GFP within condensates in the nucleus (Figure 5B, left, yellow arrow). PCP-CFP was also co-localized in some condensates, indicating that the reporter expression was enhanced in condensates. In contrast, when dSM- or NM-containing 12 × PP7 reporter plasmids were transfected, SOX2 and p300 were diffused in the nucleus without reporter expression (Figure 5B, middle and right). These observations indicated that enhancer activity was correlated with co-condensation of SOX2 and p300 (Figure 5C). With SM-containing reporter, Pearson's coefficients between mCherry signal and CFP or GFP signals were 0.53 and 0.67, respectively, while those values were 0.37 and 0.22 or 0.49 and 0.29 with dSM- or NM-containing reporter, respectively, showing that SOX2 and p300 were well co-localized at SM for transcriptional activation (Figure 5D).

To investigate if PCP-CFP was enriched on SOX2 condensates, we performed a time-course confocal analysis by transfecting PCP-CFP plasmid at 72-hour post-transfection of SOX2-mCherry and SM-containing reporter plasmid (Supplementary Figure 10C, left). CFP signals at SOX2 condensates were increased time-dependently, demonstrating that transcription occurred at pre-formed SOX2 condensates (Supplementary Figure 10C, right). To further characterize properties of condensates, we performed FRAP assays in MEF cells co-transfected with PCP-CFP, SOX2-mCherry, p300-GFP and the 12 × PP7-SM reporter plasmid. After photo-bleaching, mCherry signals in conden sates exhibited an immediate recovery with Tau of 11.4 s (Figure 5E), indicating that condensates exhibited liquid-like properties. Additionally, we treated cells with 1,6-hexanediol and observed dissolution of condensates (Supplementary Figure 10D). Taken together, these findings highlight the importance of SOX2 condensates for transcriptional activation of mSE078 and provide evidence for liquid-like droplet properties of condensates.

We then assessed detailed mechanisms of how SOX2:p300 co-condensation transcriptionally activated mSE078. As SOX2 facilitated both p300-mediated chromatin acetylation and *trans*-autoacetylation of p300 in this study, we hypothesized that SOX2:p300 co-condensation could further enhance these reactions in condensates. When we tested p300 HAT activity toward histone octamers with increasing PEG8,000 concentration, we observed that p300 HAT activity decreased along with PEG8000, consistent with the behavior of p300 in a previous study (Supplementary Figure 11A) (75). Therefore, we conducted experiments by varying the concentration of glycerol, which also induced SOX2 condensation in the IVA reaction containing SOX2-Cy3, p300-AF430 and chromatin, although the condensation-inducing activity was lower than that of PEG8000 (Supplementary Figure 11B). As shown in Supplementary Figure 11C, western blot analysis revealed that chromatin acetylation was augmented with increasing glycerol concentrations in the presence of SOX2, whereas p300 alone could not acetylate chromatin under the same conditions (Supplementary Figure 11D). Next, time-course analysis of *trans*-autoacetylation of p300 (K1499ac) revealed that *trans*-autoacetylation of p300 was highly enhanced by glycerol, indicating that SOX2:p300 co-condensation was critical for activating p300 in transcriptional condensates at mSE078 (Supplementary Figure 11E).

To further validate the effect of co-condensation on p300-mediated chromatin acetylation, we examined levels of chromatin acetylation in co-condensation of the SOX2:p300:chromatin after treatment with 1,6-HD. Condensation and acetyltransferase activity assays confirmed that 1,6-HD (5%) inhibited condensation by 40% (Supplementary Figure 12A, B) and impaired chromatin acetylation in a concentration-dependent manner in the presence of 10% glycerol (Supplementary Figure 12C). However, we did not observe a significant decrease in p300 HAT activity in the presence of 1,6-HD at concentrations of up to 5% (Supplementary Figure 12C). Consistent with the effect of 1,6-HD on condensation in MEF cells (Supplementary Figure 10A, B), these results strongly suggest that hydrophobic interaction is critical for co-condensation of SOX2 and p300, which is essential for enhancing p300-mediated chromatin acetylation. This observation was also consistent with reduced chromatin acetylation and SOX2:p300:chromatin co-condensation when SOX2 mutants lacking the binding activity with p300 (E78A and E86A) were introduced (Supplementary Figures 9D, 12D and E). Similar results were shown in MEF cells co-transfected with mCherry-labeled SOX2 mutants, PCP-CFP and p300-GFP (Supplementary Figure 13). Discrete condensates of PCP-CFP were observed only in cells co-transfected with wild-type SOX2 (Supplementary Figure 13, wild-type). These findings suggest that specific interactions between SOX2 and p300 are essential for transcriptional activation of SM-containing plasmid. Interestingly, in MEF cells co-transfected with mutant SOX2-mCherry, formation of SOX2-independent condensates by p300 was observed, similar to what was observed when p300 was transfected alone (Supplementary Figure 13, E78A and E86A). This suggests that the loss of specific SOX2:p300 interaction resulting from SOX2 mutations can lead to the dissolution of SOX2:p300 co-condensates. Collectively, these results prove that SOX2:p300 co-condensation can lead to transcriptional activation of mSE078 by facilitating *trans*-autoacetylation of p300 and p300-mediated chromatin acetylation.

### SOX2-dependent regulation of MKRN1 expression in cancer and stem cells

**Figure 6.**
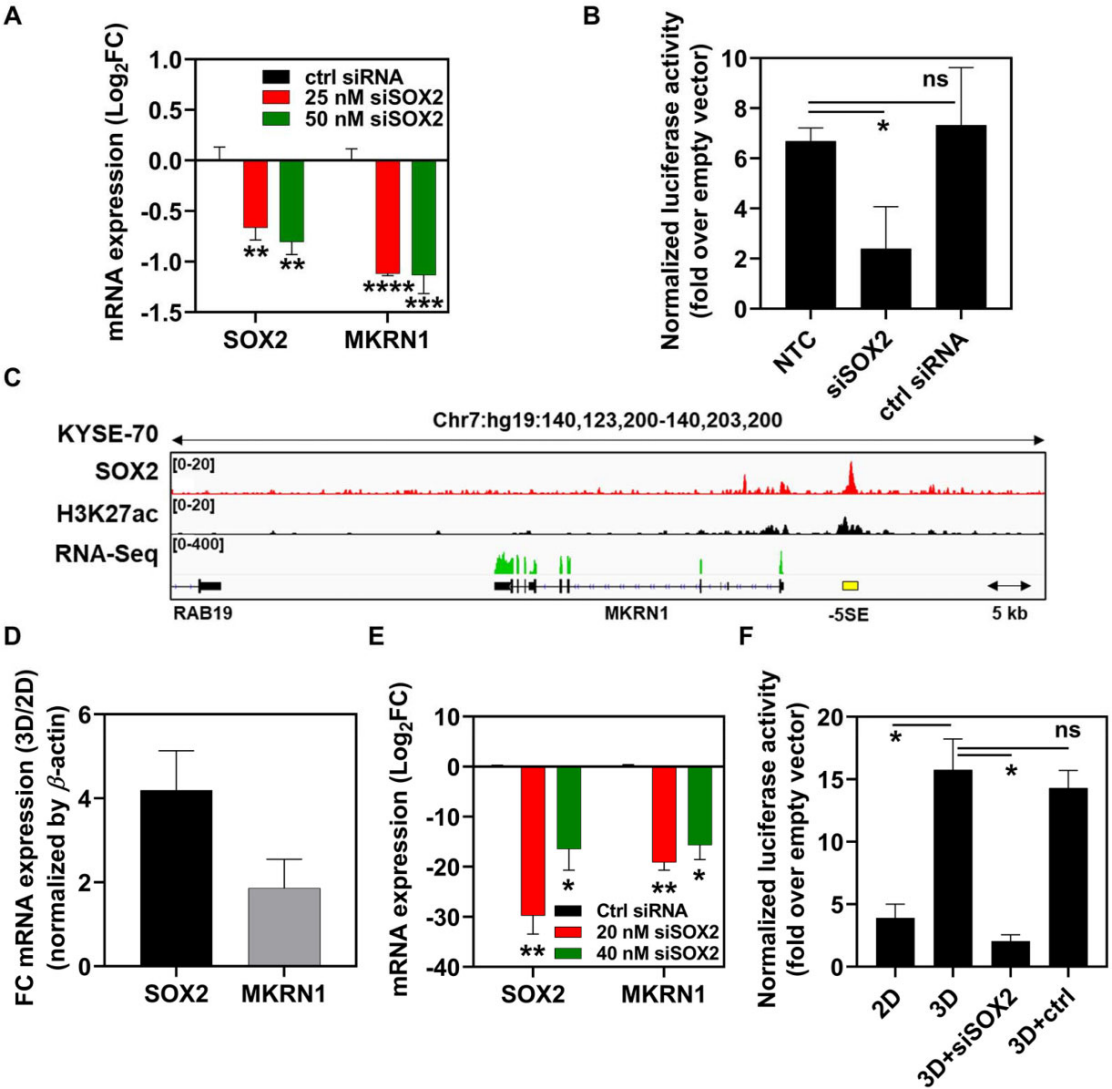
Quantitative RT-PCR (qRT-PCR) and luciferase assay in OE33 cells. (**A**) qRT-PCR analysis of mESCs 24 h post-transfection of SOX2 siRNA (siSOX2) or negative control siRNA. Log_2_FC (Fold change) mRNA expression levels (control siRNA versus siSOX2) were calculated using the comparative threshold cycle (*ΔΔ*Ct) method. (**B**) Luciferase activity of pGL3-SM in non-transfected cells (NTC), siSOX2- and control siRNA-treated mESCs. (**C**) ChIP-seq profiles (raw reads) of SOX2, H3K27ac and RNA-seq (raw reads) at *MKRN1* region in cells of human esophageal squamous cell carcinoma cell line (KYSE-70). -5SE is indicated as a yellow bar. (**D**) qRT-PCR analysis in 2D and 3D-cultured OE33 cells. SOX2 and MKRN1 mRNA levels were normalized to that of *β*-actin. FC mRNA expression levels (2D versus 3D) were calculated as described above. (**E**) qRT-PCR analysis of 3D-cultured OE33 cells 24 h post-transfection of SOX2 or negative control siRNA. (**F**) Luciferase activity of pGL3 luciferase reporter plasmid containing -5SE and human *MKRN1* core promoter in 2D-, 3D-cultured and siSOX2-treaed 3D-cultured OE33 cells. All luciferase activities were normalized to Renilla luciferase activity from co-transfected pRL-TK plasmid. Their relative values to the activity of empty pGL3 reporter are shown. Error bars indicate SEMs of three independent experiments. *P*-values were calculated using a two-tailed *t*-test. (ns) *P* < 0.1234; (*) *P*< 0.0332; (**) *P* < 0.0021; (***) *P*< 0.0002; (****) *P*< 0.0001.

In this study, we found that mSE078 was a potent *MKRN1* SE in mESCs. To validate the role of SOX2 in the regulation of *MKRN1* expression in mESCs through mSE078, we monitored MKRN1 mRNA level and enhancer activity of mSE078 (SM) before and after SOX2 knockdown by RNA interference. SOX2 knockdown decreased mRNA levels of both SOX2 and MKRN1 in mESCs (Figure 6A) and abolished enhancer activity of SM in mESCs (Figure 6B). These results demonstrated that MKRN1 expression in mESCs was highly SOX2-dependent.

MKRN1 is highly associated with tumorigenesis and cancer stemness as an E3 ubiquitin ligase (81). We validated the role of mSE078 and SOX2 in esophageal cancer. Although the expression of SOX2 and MKRN1 in esophageal cancer is known (82–84), studies on their correlation have not been reported yet. First, from genomic analysis using previous ChIP-seq data in human ESCs, we found -5SE corresponding to mSE078 in mESCs (Supplementary Figure 14A) (85,86). Consistent with mSE078 in mESCs, -5SE in human ESCs was highly enriched in MED1, p300 and H3K27ac. It has also been revealed that -5SE is a constituent enhancer of SE_02_03601426 in human ESCs (87). Similarly, genomic analysis using previous ChIP-seq data showed that -5SE was found in a human esophageal cancer cell line (KYSE-70) (Figure 6C). Based on the read threshold 12 for SOX2 ChIP-seq in KYSE-70 cells set by the tangent line of slope 1 in a scaled plot of SOX2 ChIP-seq read to ranked order by increasing value, we noticed that -5SE had an enriched SOX2 binding in human esophageal cancer (Supplementary Figure 14B). We then prepared OE33 cells, a human esophageal adenocarcinoma cell line, by three-dimensional (3D) culture to enhance cancer stem cells (CSC) properties. The enhancement was confirmed by expression of aldehyde dehydrogenase (ALDH), a CSC marker, by FACS (Supplementary Figure 14C, D) (84). Under these conditions, mRNA levels of SOX2 and MKRN1 were increased 4- and 2-folds, respectively (Figure 6D). In contrast, SOX2 knockdown via RNA interference significantly reduced mRNA levels of both SOX2 and MKRN1, confirming SOX2-dependent regulation of MKRN1 expression in OE33 cells (Figure 6E).

To investigate SOX2-dependent enhancer activity of -5SE, a region showing higher SOX2 ChIP-seq reads than the read threshold (chr7:hg19:140185361–140185675) and a potent human *MKRN1* promoter were cloned into the pGL3 luciferase reporter plasmid. We found that enhancer activity of -5SE was 3-fold higher in 3D-cultured OE33 cells than in 2D-cultured cells. However, SOX2 knockdown by RNA interference significantly reduced the enhancer activity of -5SE, suggesting that enhancer activity was promoted by increased levels of SOX2 in 3D-cultured cells (Figure 6F). Furthermore, similar to enhancer activity of mSE078 (SM) in MEF cells, enhancer activity of -5SE in HEK cells was more susceptible to SOX2 than OCT4 or NANOG (Supplementary Figure 14E). According to ChIP-qPCR analysis in OE33 cells, significant losses in binding of p300 and level of H3K27ac upon SOX2 knockdown were observed, demonstrating that the working mechanism of -5SE was highly similar to that of mSE078 (Supplementary Figure 14F). SOX2 knockdown also reduced the binding of p300 and RNA Pol II and the level of H3K27ac at MKRN1 promoter (Supplementary Figure 14G). From GeneHancer database (88), -5SE was predicted to be linked to MKRN1 gene. Their physical interaction has been confirmed by RNA Pol II ChIA-PET data in HeLa S3 cells (GSM970211) in GEO database (Supplementary Figure 15) (89). Overall, these results together support that -5SE works as an SE of MKRN1 in human cells. Both MKRN1 expression and enhancer activity of -5SE were largely perturbed by changing the cellular level of SOX2, indicating that transcriptional activation of SOX2-enriched SEs was highly dependent on SOX2 concentration, consistent with our *in vitro* analysis of mSE078.

## Discussion

**Figure 7.**
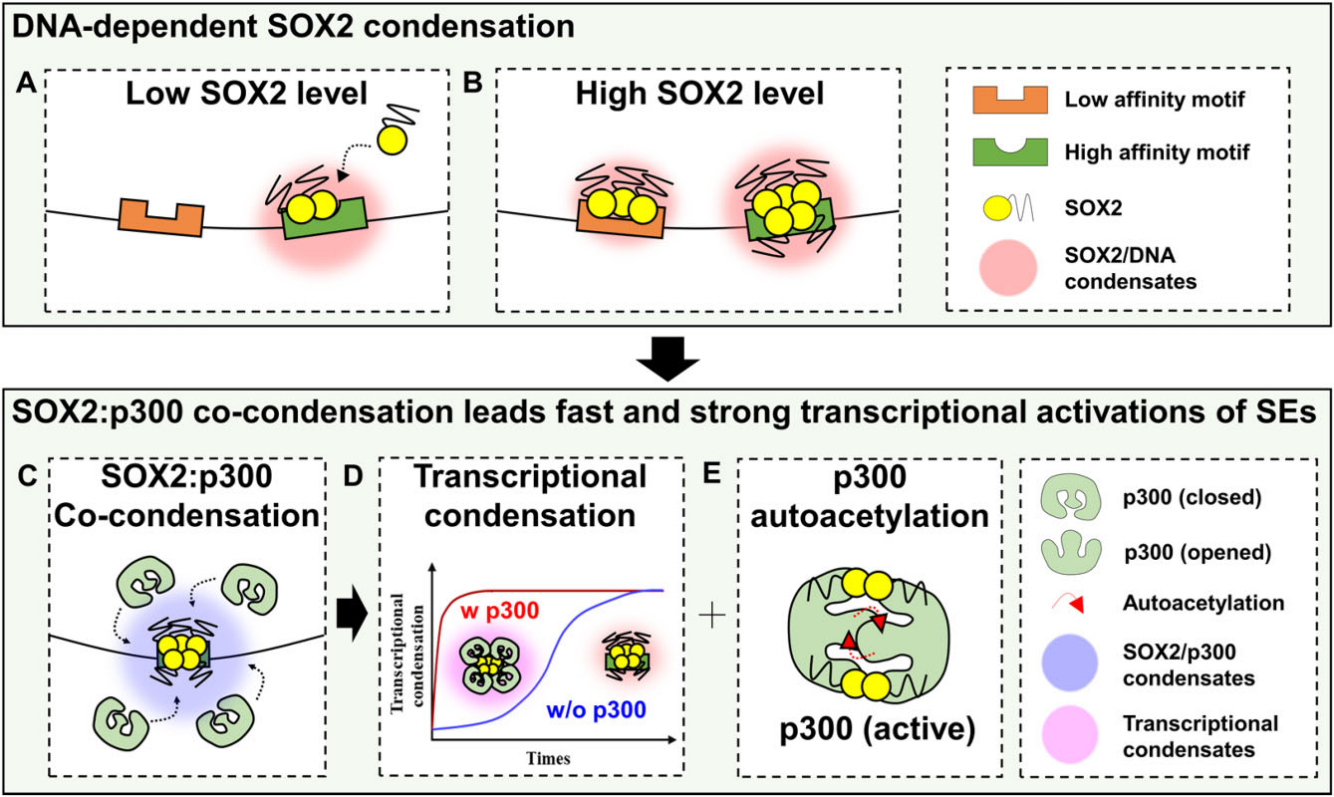
Models of condensation-dependent transcriptional activation of SEs. (**A**) When the level of SOX2 is low, SOX2 condenses on DNA with a high affinity motif *via* its specific DNA binding activity. (**B**) When the SOX2 level is high, condensation occurs at both low and high affinity motifs. (**C**) DNA-bound SOX2 recruits p300 into SOX2:DNA condensates and forms SOX2:p300 co-condensation. In condensates, (**D**) p300 accelerates the formation of transcriptional condensation by co-condensation with SOX2. Moreover, (**E**) SOX2 facilitates *trans*-autoacetylation of p300, which leads to activation of catalytic activity of p300, thereby achieving a fast and strong transcriptional activation of SEs.

Since SEs have been recognized as key regulators of gene expression, particularly in cell fate determination and cancer, extensive research has been conducted to understand the components, functions, action mechanisms and disease relevance of SEs. While the importance of pioneer transcription factors (pTFs) in initiating transcription at SEs is well-established, there is still a limited understanding of how pTFs precisely mediate transcriptional activation of SEs at the molecular level. In our study, we focused on the interaction between SOX2 and mSE078 SE as a model system to investigate molecular mechanisms underlying pTF-mediated transcriptional activation of SEs (Figure 7). Based on our experimental evidence, we propose the following mechanism: SOX2 binds to and forms condensates with the SOX2-binding motif within SEs. These SOX2:SE condensates further recruit p300 through direct interactions between SOX2 and p300. The recruitment of p300 leads to chromatin acetylation and subsequent transcriptional activation of SEs. However, it is important to note that SOX2 is associated with various other components within SEs, including other transcription factors, coactivators and enhancer RNAs (18,90–92). Synergistic effects of these SE components, along with SOX2, on transcriptional condensation and activation are likely to further enhance transcriptional bursting observed for SEs.

Condensation of TFs with DNA is largely affected by characteristics of their cognate binding motifs. For example, OCT4 condensation is influenced by motif features in DNA, such as motif density in SE and affinity to OCT4 (25). It has been reported that CpG methylation of the KLF4-binding motif can enhance KLF4 condensation (93). In this study, we also confirmed that SOX2 condensation is highly dependent on the affinity of the motif to SOX2 as well as SOX2 concentration (Figure 2C, D). Since it is known that SOX2 binding to its target motif is altered by the location and chemical modification of the motif (56,93,94), it is expected that transcriptional activation of SOX2-enriched SEs via SOX2 condensation in cells will be dynamically regulated by motif affinity to SOX2 in SEs and cellular concentration of SOX2. In the aspect of condensation mechanism, one should note that condensates of TF with DNA can be formed either by protein condensation on DNA surface (63) or protein monolayer formation on DNA (95). Previous studies have demonstrated that SOX2 condensates are formed at specific positions in bacteriophage λDNA and that SOX2 self-associates in a DNA-dependent manner (35,96). These characteristics of SOX2 are reminiscent of those observed for KLF4, which undergoes condensation on the DNA surface (63). However, we observed that SOX2 also formed condensates with non-specific DNA element at higher SOX2 concentration, suggesting the potential role of non-specific SOX2:DNA interactions in SOX2 condensation. These collective findings underscore the dynamic and multifaceted nature of SOX2 condensation mediated by various physical mechanisms. Consequently, the regulation of transcriptional activation of SOX2-enriched SEs is expected to be controlled in a highly intricate and sophisticated manner. Further systematic studies for measuring SOX2:DNA stoichiometry in SOX2 condensates are required to elucidate detailed mechanisms of SOX2 condensation.

Based on current results, a model of SOX2 condensation at SOX2-enriched SEs can be proposed as shown in Figure 7A and B. When SOX2 concentration is low, it mostly binds to a high-affinity motif and self-associates in a DNA-dependent manner to form SOX2:DNA condensates (Figure 7A). This can be supported by current EMSA results (Figure 2B), condensation assay (Figure 2C) and a previous study (96). This model was consistent with our reporter assay, in which the reporter plasmid containing a higher-affinity motif produced a strong enhancer activity in mESCs (Figure 2F). However, when SOX2 concentration is high, SOX2 binds and forms condensates at both high- and low-affinity motifs, resulting in transcriptional activation at both motifs (Figure 7B). Indeed, although we found no enhancer activity from the non-SOX2 binding region of mSE078 (NM) in MEF cells with ectopic SOX2 expression (Figure 2F), a stronger SOX2 expression than that used in this study produced a certain enhancer activity of NM (data not shown). These condition-dependent properties of SOX2 condensation could be a mechanism by which cells dynamically switch SE activity according to environmental cues, which might explain the dose-dependent regulatory function of SOX2 (47,97). Interestingly, we observed that SOX2 droplets is not found in SOX2-expressing MEF cells but in mESCs (Supplementary Figures 6D and 10A), suggesting the presence of the cellular context-dependent ability of SOX2 condensation. Consistently, recent study about the lack of SOX2 droplets in MEF also supports this interpretation (72).

SOX2 and p300 are known to cooperate in gene regulation. However, detailed mechanisms of their association and their functional implications in transcriptional condensation and activation remain unclear (18,54,98). Previous studies have shown that p53 interacts with p300 through various domains, leading to structural changes in p300 (68,99). In the current study, we demonstrated that SOX2 directly interacted with p300 and formed co-condensates in a DNA-dependent manner (Figure 3). Although we do not have direct molecular evidence for structural changes of p300 upon SOX2 binding, it is reasonable to speculate that structural changes in p300 similar to those in p53:p300 complex may occur in the SOX2:p300 complex since SOX2 also interacts with p300 through different p300 domains including at least KIX and bromodomain (Supplementary Figure 6C). Further studies on the structure of SOX2:p300 complex are required to understand the binding mode of p300 and SOX2 and the structural basis underlying SOX2-dependent p300-mediated chromatin acetylation and autoacetylation of p300.

Transcriptional condensation is highly associated with strong and fast transcriptional activations (23,25,34). This is also evident in our data. We observed that the cellular distribution of p300 was significantly altered by ectopic expression of SOX2 (Supplementary Figure 6D). In addition, SOX2 and p300 formed co-condensates with SOX2-enriched SE, which was highly DNA-dependent and affected by both specific and non-specific interactions between SOX2 and p300 (Figure 5B, Supplementary Figures 10 and 13). We demonstrated that SOX2:p300 co-condensation accelerated the formation of transcriptional condensates (Figure 3I, Supplementary Figure 7, Figure 7C, D). At the same time, we found that SOX2:p300 co-condensation enhanced *trans*-autoacetylation and, thus, the catalytic activity of p300 (Figure 7E, Supplementary Figure 11C, E). Furthermore, unlike condensates of p300 alone, which were dissolved by autoacetylation of p300 (75), SOX2:p300 co-condensates stably existed regardless of p300 autoacetylation (Supplementary Figure 11F). These data demonstrate that SOX2 can recruit p300 into SOX2 condensates to dynamically regulate genes of SOX2-enriched SEs and that SOX2:p300 co-condensation is necessary not only for transcriptional activation of SOX2-enriched SEs, but also for maintaining their activities. They are highly consistent with the fast and robust transcription observed with p65:p300 co-condensation (34). However, although our current data support the role of SOX2:p300 co-condensation in transcription activation of SOX2-enriched SEs, we acknowledge the need for additional experiments to provide direct evidence for the existence of SOX2 condensates in SEs and their roles in transcription activation in cells. Further experiments are required to validate our model.

Regulation of SE activity has attracted much interest as a therapeutic strategy because SEs are highly relevant to many critical diseases (100). For example, small molecules targeting key components of SEs, such as BRD4 (101), p300 (102) and CDK7 (103), have been studied in multiple myeloma, autoimmune diseases and cancer drugs, respectively. Similarly, we proved the contribution of SOX2 to cancer stemness via SOX2-enriched SE in esophageal cancer (Figure 6). Disruption of the specific interaction between SOX2 and p300 resulted in perturbation of SOX2:p300 co-condensation and inhibition of transcriptional activation of SOX2-enriched SE (Supplementary Figures 12D, E, 13). These results suggest that p300 is a key coactivator of SOX2-enriched SEs and that their activity can be regulated by targeting the specific interaction between SOX2 and p300. Presence of many SOX2-enriched SEs near stemness genes highlights the potential of targeting the SOX2-dependent transcriptional activation process for controlling cell differentiation and cancer stemness (Supplementary Figure 1). Similar strategies have been explored in other systems such as inhibition of SNAIL-p300 (104), BET proteins-NEUROD1 (105) and YAP-TEAD4 (106) as promising anticancer approaches. Considering that SOX2 is generally considered ‘undruggable’, findings of our study provide valuable insights for the development of novel therapeutic strategies. By focusing on inhibiting the direct interaction between SOX2 and p300, it may be possible to design or screen chemical compounds that can disrupt this interaction, thereby interfering with the condensation process and modulating transcriptional activities of SEs. Such approaches could have therapeutic potential for controlling cell fate determination, suppressing cancer stemness and potentially treating diseases associated with dysregulated SOX2-p300 signaling. Further research and drug development efforts are necessary to explore the feasibility and efficacy of targeting the interaction between SOX2 and p300 for therapeutic purposes. The potential of this approach offers new opportunities for intervening SOX2-dependent transcriptional regulation. It might pave the way for future therapeutic interventions.

## Supplementary Material

gkad908_supplemental_file

## Data Availability

The data underlying this article will be shared on reasonable request to the corresponding author.
