# Supplementary material for: Molecular basis for SOX2-dependent regulation of super-enhancer activity
Source: Nucleic Acids Res. 2023 Nov 1;51(22):11999–2019. doi: 10.1093/nar/gkad908 (PMC10711550; doi:10.1093/nar/gkad908)
Supplement: gkad908_supplemental_file [file gkad908_supplemental_file.docx]

**Supplementary Figures and Tables**

**Molecular basis for SOX2-dependent regulation of super-enhancer activity**

Wanki Yoo^1^, Yi Wei Song^1^, Jihyun Kim^2^, Jihye Ahn^2^, Jaehoon Kim^2^, Yongdae Shin^3^, Je-Kyung Ryu^3^, and Kyeong Kyu Kim^1^*

^1^Department of Precision Medicine, Graduate School of Basic Medical Science (GSBMS), Institute for Antimicrobial Resistance Research and Therapeutics, Sungkyunkwan University School of Medicine, Suwon 16419, Republic of Korea

^2^Department of Biological Sciences, Korea Advanced Institute of Science and Technology, Daejeon 34141, Republic of Korea

^3^Department of Mechanical Engineering, Seoul National University, Seoul, 08826, Republic of Korea

^3^Department of Physics & Astronomy, Seoul National University, Seoul, 08826, Republic of Korea

**
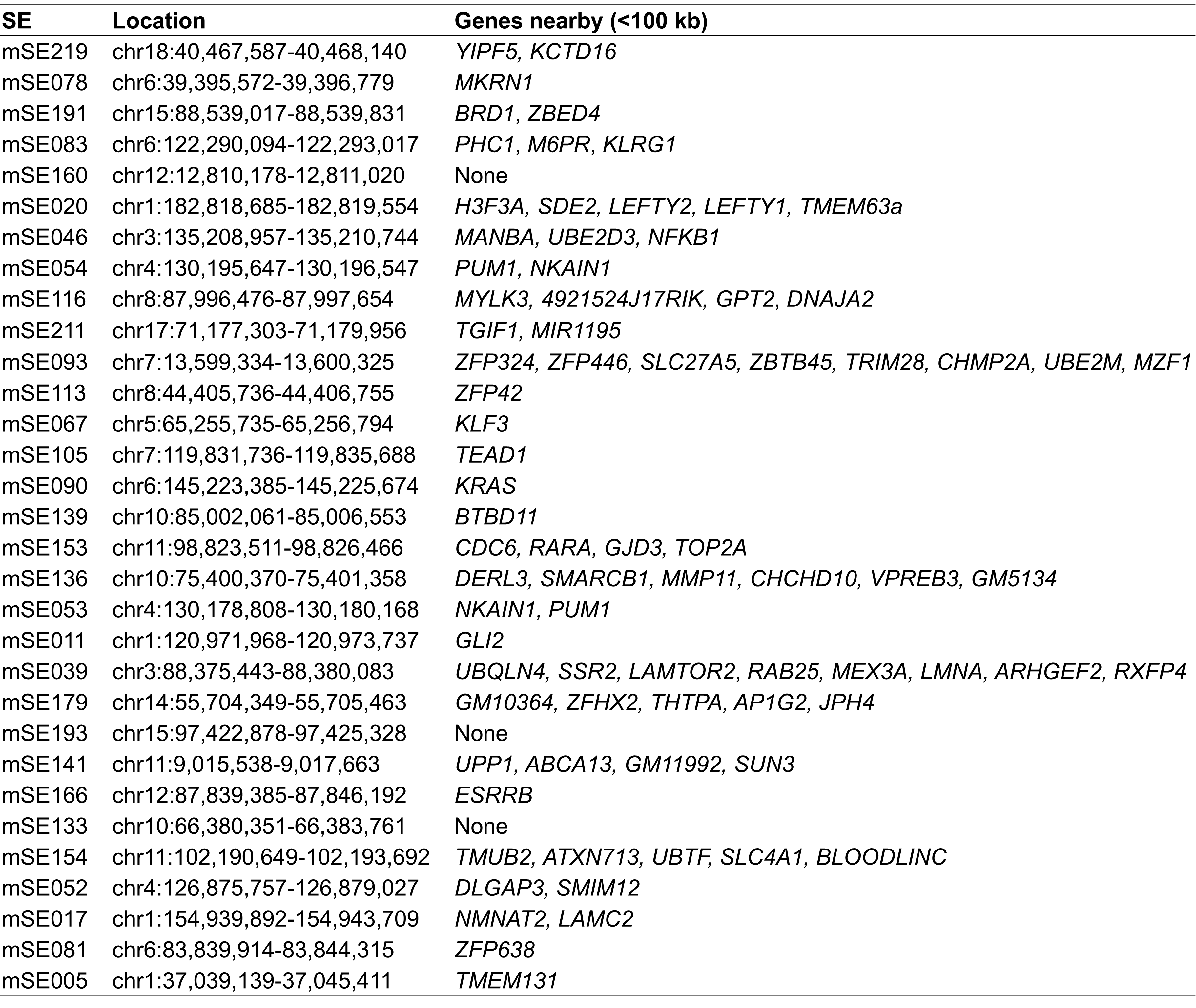
Supplementary Figure 1. List of SOX2-enriched SEs and heat maps of total SEs in mouse ESCs.** List of 31 SOX2-enriched SEs. Genomic location and genes nearby (within 100 kb from the center of SE) are shown.

**
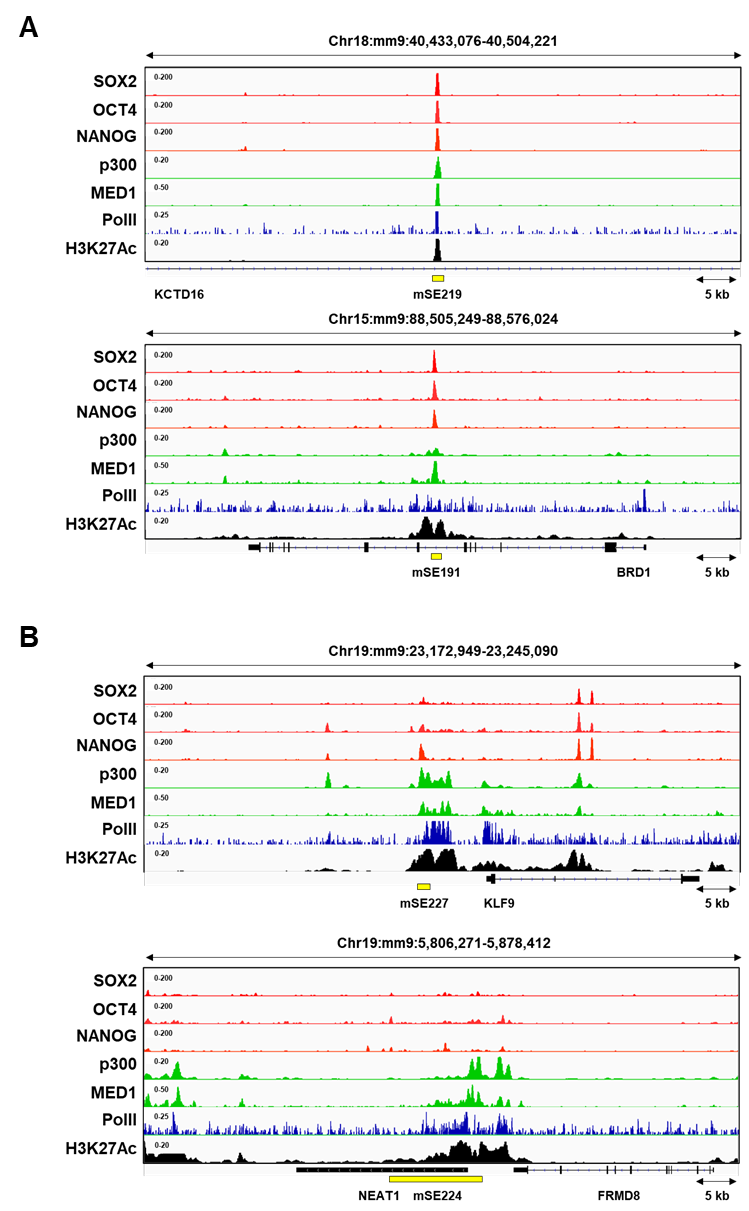
**

**Supplementary Figure 2. ChIP-seq profiles of other SOX2-enriched SEs and non-SOX2-enriched SEs in mESCs.** (**A**) ChIP-seq profiles of other SOX2-enriched SEs. mSE219 (top) and mSE191 (bottom) were ranked as 1^st^ and 3^rd^ highest SOX2-enriched SEs, respectively. (**B**) ChIP-seq profiles of non-SOX2-enriched SEs. mSE227 (top) and mSE224 (bottom) were ranked as the last with 0 SOX2-binding density.

**
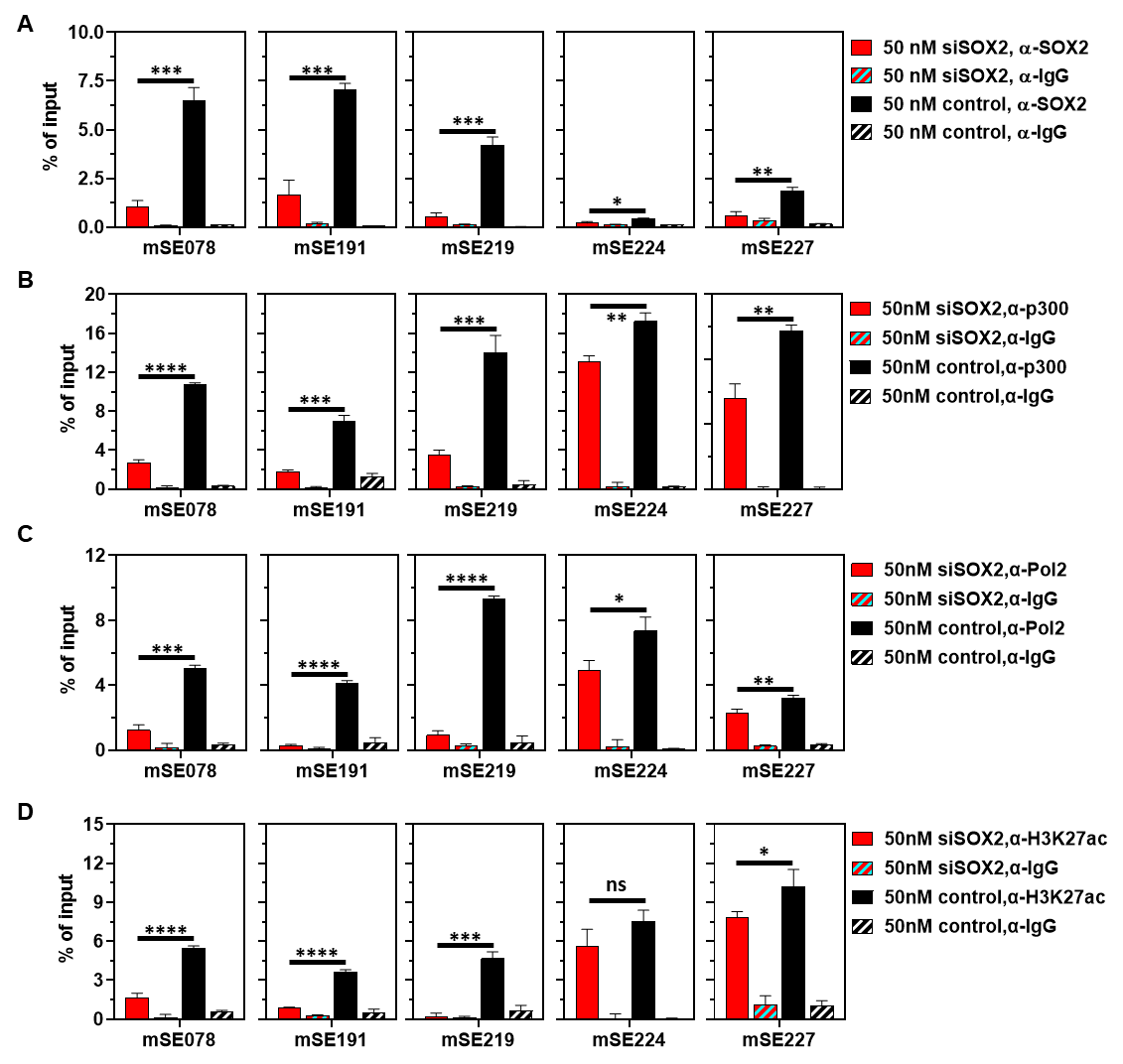
Supplementary Figure 3. Chromatin immunoprecipitation followed by quantitative PCR (ChIP-qPCR) analysis in mESCs.** (**A-D**) qPCR analysis of each SEs. ChIP was done with anti- (**A**) SOX2, (**B**) p300, (**C**) Pol2, or (**D**) H3K27ac antibody. For SOX2 knockdown, mESCs were inoculated at 24-well plate 24 hours before transfection. At the day of transfection, final 50 nM siRNA or negative control siRNA was treated to mESCs and further incubated for 24 hours in CO_2_ incubator at 37 °C. ChIP-qPCR was done as described in materials and methods. The enrichment was shown relatively to input. *P*-values were calculated using a two-tailed *t*-test. (ns) *p* < 0.1234; (*) *p* < 0.0332; (**) *p* < 0.0021; (***) *p* < 0.0002; (****) *p* < 0.0001.

**
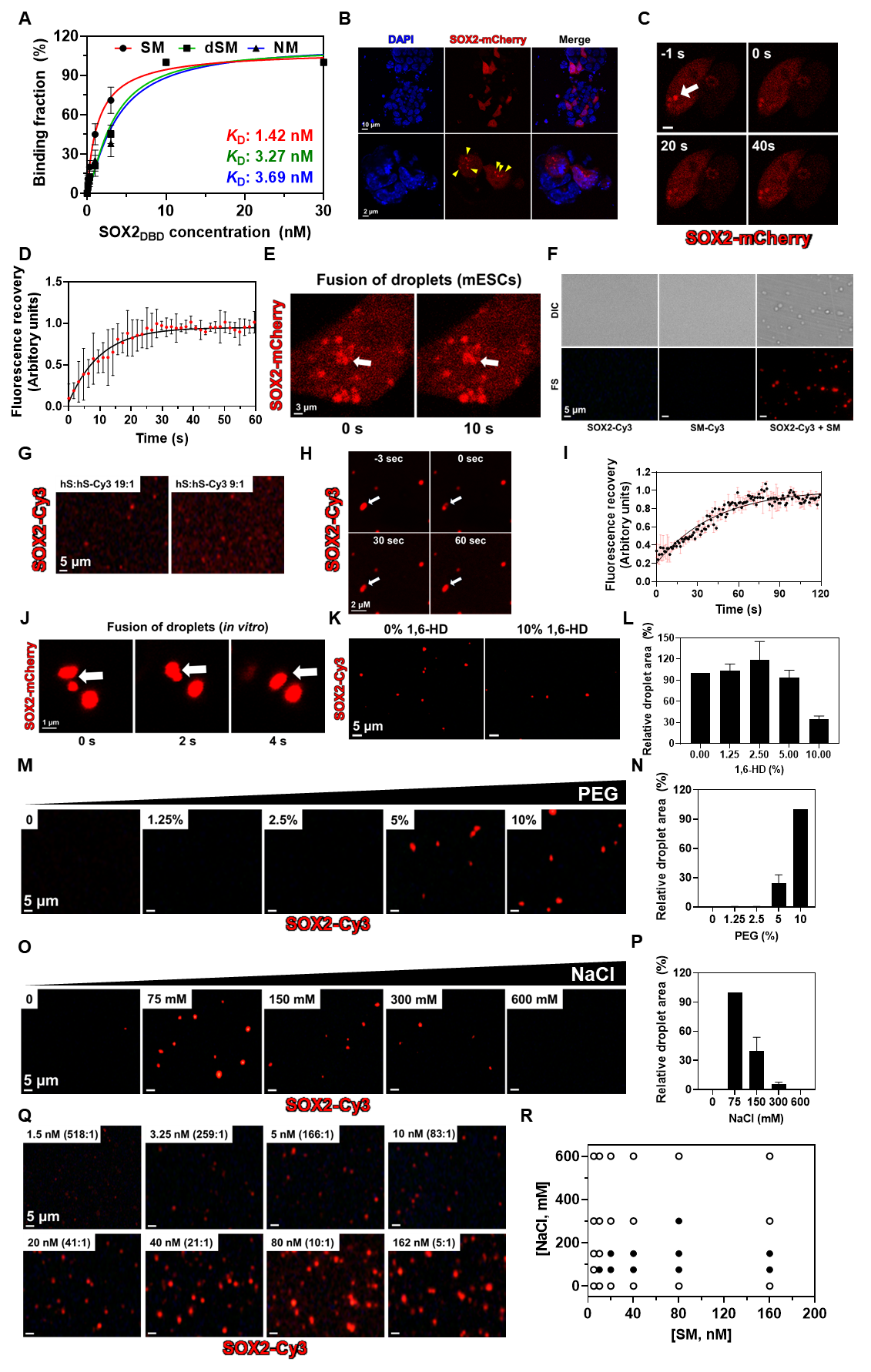
**

**Supplementary Figure 4. SOX2 condensation in various conditions.** (**A**) A plot of SOX2_DBD_ binding on SM, dSM, and NM to protein concentration. Total binding fraction was calculated by quantifying the decrement of free DNA band with increasing SOX2_DBD_ in Figure 2B. (**B**) Confocal microscopic analysis of SOX2-mCherry expression in mESCs. Top: 40X magnification, bottom: close-up images. mCherry foci were indicated by yellow arrow. (**C**, **D**) Fluorescence recovery after photobleaching (FRAP) analysis of SOX2 condensates in mESCs. Photobleaching was initiated at 0 s. Curve shows mean (red dot) and SEM (black bar) of Cy3 intensity of 15 droplets. The FRAP recovery curves were fitted to the double exponential function. (**E**) Fluorescence images showing droplet fusion (white arrow) in mESCs expressing SOX2-mCherry. (**F**) Differential interference contrast (DIC) and fluorescence (FS) microscopic analysis of SOX2 condensation. (**G**) FS microscopic analysis of SOX2 condensation with SM with the ratio of unlabeled SOX2:SOX2-Cy3 at 19:1 (left) and 9:1 (right). (**H, I**) Fluorescence recovery after photobleaching (FRAP) of droplets measuring SOX2-Cy3. Photobleaching was initiated at 0 s. Curve shows mean (red dot) and SEM (black bar) of Cy3 intensity of 10 droplets. The FRAP recovery curves were fitted to the double exponential function. (**J**) Fluorescence images showing droplet fusion of SOX2-Cy3 (white arrow) *in vitro*. (**K**) FS microscopic analysis of SOX2 condensation with SM in the presence of 1,6-hexandiol (1,6-HD) and (**L**) relative total area of droplets. Values are relatively shown to the total area of droplets at 0% 1,6-HD. FS microscopic analysis of SOX2 condensation with SM in different PEG (**M**, **N**) or NaCl (**O**, **P**) concentrations. Relative values to the total area of droplets with 10% PEG or 75 mM NaCl are shown in (**N**) or (**P**), respectively. Error bars are standard error of the mean (SEM) of two independent experiments. All samples were visualized after 1 hour incubation at room temperature. Relative total area of droplets was quantified as described in materials and methods. (**Q**) FS microscopic analysis of SOX2 condensation with different concentration of SM. The representative fluorescent images are shown. SOX2-Cy3 (837 nM) was incubated with SM in PS buffer (20 mM Tris-HCl, 150 mM NaCl, 5 mM MgCl_2_, 10% PEG8,000). The concentration of SM and SOX2:SM ratio are indicated in the images. After 1 hour incubation, the images were analyzed. (**R**) Droplet formation at different NaCl and SM concentrations. Solid and open circles represent the formation and the non-formation of droplets, respectively. If the mean fluorescence intensity was higher than 0.3, images were considered as formation of droplets. The value was determined using ImageJ software. *P*-values were calculated using a two-tailed *t*-test. (ns) *p* < 0.1234; (*) *p* < 0.0332; (**) *p* < 0.0021; (***) *p* < 0.0002; (****) *p* < 0.0001. In all the experiments, SOX2-Cy3 (837 nM) and SM-Cy3 (150 nM) were used.

**
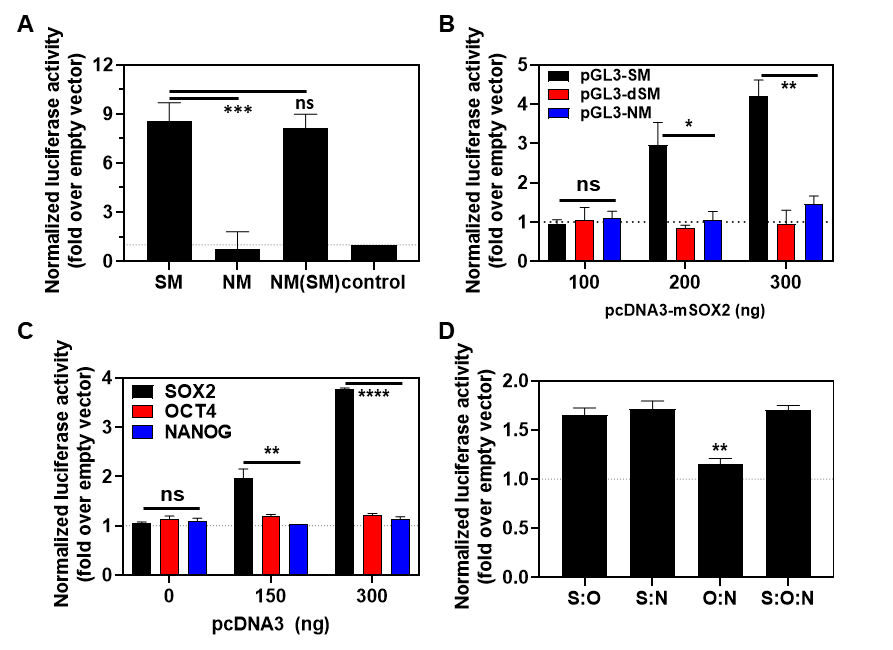
Supplementary Figure 5. Luciferase assay of pGL3-SM, -NM, and NM(SM) in mESCs.** (**A**) Luciferase activity of pGL3-SM, -NM, or -NM(SM) in mESCs. (**B**) Luciferase activity of pGL3-SM, dSM and NM in MEF cells with the expression of SOX2. 100–300 ng of pcDNA3-mouse SOX2 (mSOX2) were co-transfected to MEF cells with pGL3 luciferase reporter vectors. (**C**) Luciferase activity of pGL3-SM in MEF cells with the expression of SOX2, OCT4, or NANOG through co-transfection of pcDNA3-SOX2, OCT4 or NANOG with pGL3-SM vector. (**D**) Luciferase activity of pGL3-SM in MEF cells with co-overexpression of SOX2:OCT4 (S:O), SOX2:NANOG (S:N), OCT4:NANOG (O:N) and SOX2:OCT4:NANOG (S:O:N). All luciferase activities were normalized to Renilla luciferase activity from co-transfected pRL-TK plasmid, and their relative values to the activity of empty pGL3 reporter vector are shown. Error bars are standard error of the mean (SEM) of three independent experiments. *P*-values were calculated using a two-tailed *t*-test. (ns) *p* < 0.1234; (*) *p* < 0.0332; (**) *p* < 0.0021; (***) *p* < 0.0002; (****) *p* < 0.0001.

**
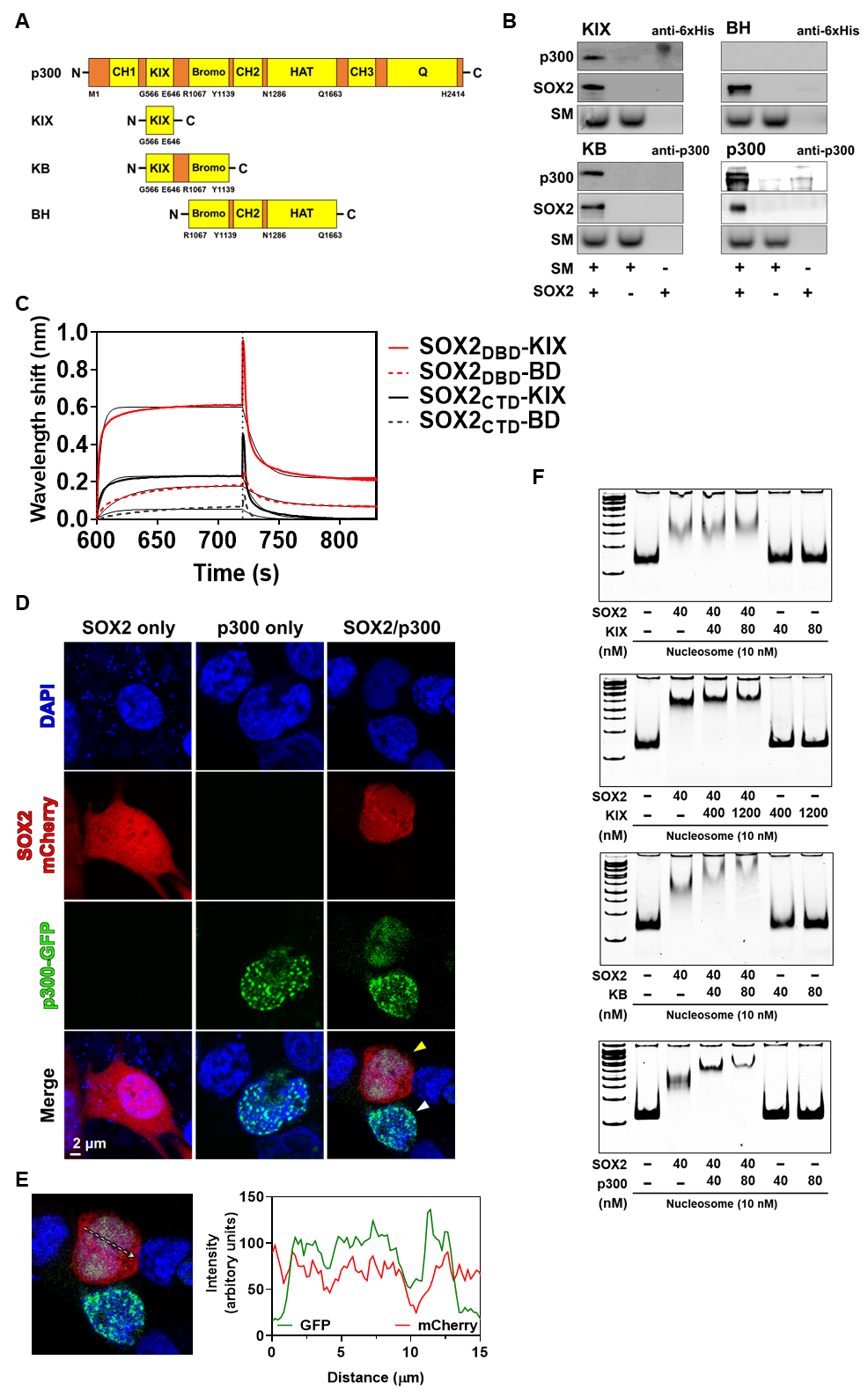
**

**Supplementary Figure 6. Pull-down assay and bio-layer interferometry (BLI) analysis.** (**A**) Schematic representations of the structures of a full length p300. (**B**) Western blot analysis of pull-down assay. SOX2 and biotinylated SM were incubated with p300 or p300 domains [KIX (aa 566–646), KB (aa 566–1139), and BH (aa 1067–1663)], followed by pulling down using streptavidin magnetic beads. (**C**) BLI analysis of SOX2_DBD_ and SOX2_CTD_ with KIX and bromodomain (BD). (**D**) Confocal microscopic analysis of MEF cells expressing SOX2-mCherry and p300-GFP. Cell expressing both SOX2-mCherry and p300-GFP and cell expressing p300-GFP alone are indicated with yellow and white arrow, respectively. (**E**) Line profile of GFP and mCherry signals in MEF cells expressing both p300-GFP and SOX2-mCherry. (**F**) EMSA of SOX2 and Nucleosomal SM in the absence or presence of p300 variants. For the samples containing p300 variants, p300 variants were added to pre-incubated samples, and the samples were further incubated for 30 minutes before running on the gel.

**
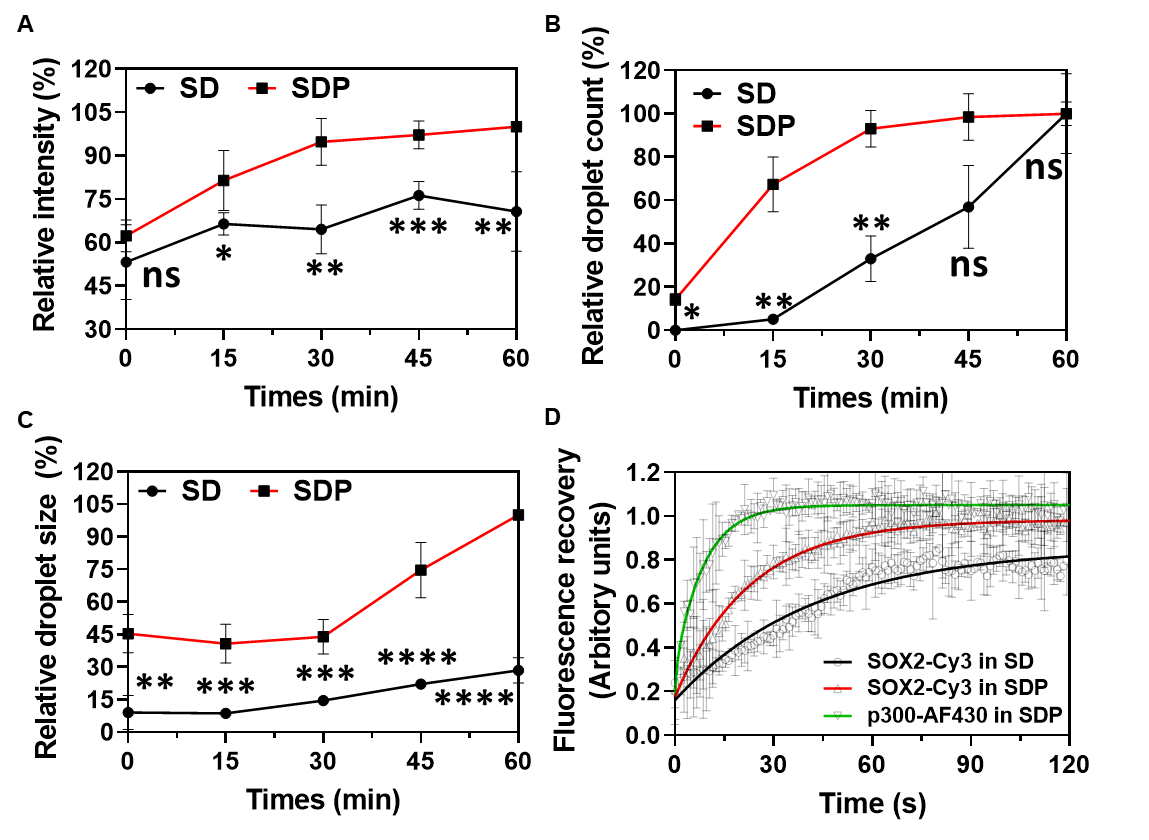
Supplementary Figure 7. Comparison of droplets of SOX2:SM (SD) and SOX2:SM:p300 (SDP).** (**A**) Relative droplet fluorescence intensity, (**B**) Relative droplet count, and (**C**) Relative droplet size was analyzed using ImageJ software. Relative values to the maximum are shown. (**D**) Fluorescence recovery after photobleaching (FRAP) of droplets of SD and SDP. Error bars are standard error of means (SEM) of 10 droplets. The FRAP recovery curves were fitted to the double exponential function. Tau values for SOX2-Cy3 in SD, SOX2-Cy3 in SDP, and p300-AF430 in SDP are 42.3 s, 22.9 s, and 8.6 s, respectively. *P*-values were calculated using a two-tailed *t*-test. (ns) *p* < 0.1234; (*) *p* < 0.0332; (**) *p* < 0.0021; (***) *p* < 0.0002; (****) *p* < 0.0001.


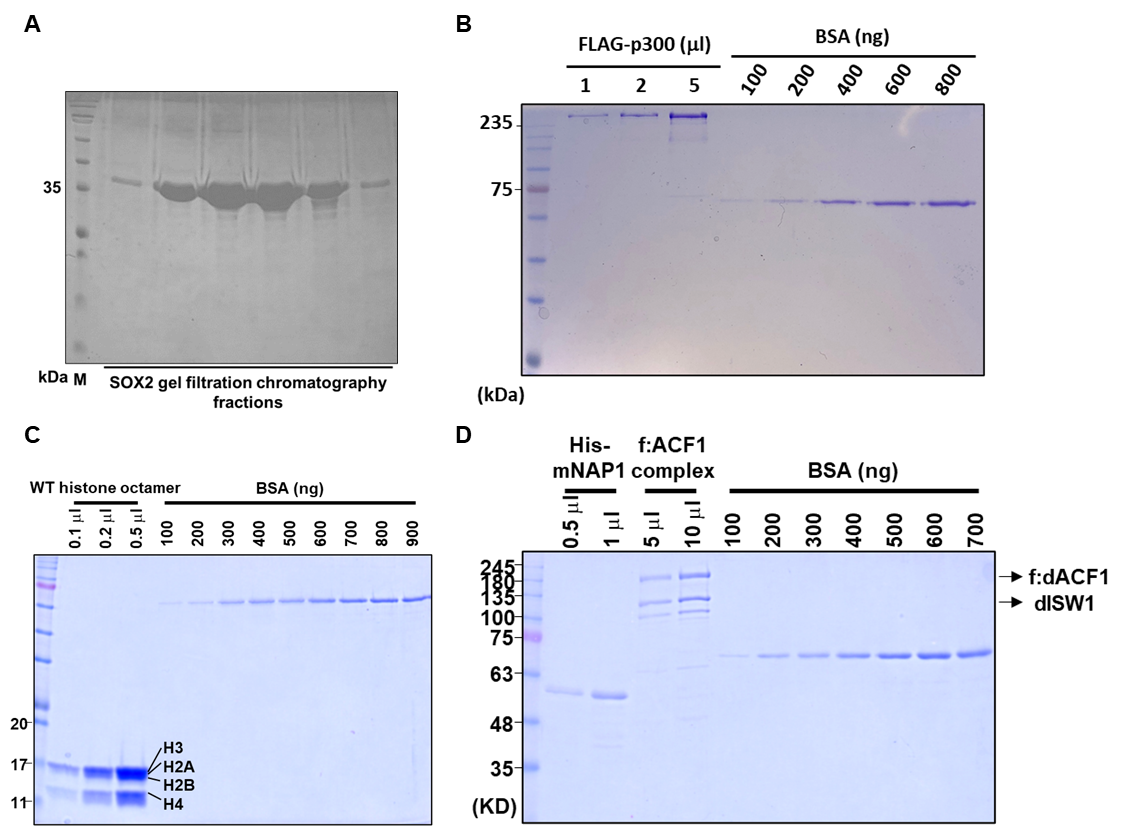
**Supplementary Figure 8.** SDS-PAGE of proteins used in chromatin assembly and *in vitro* acetylation assay. SDS-PAGE analysis of (**A**) human SOX2, (**B**) flag-tagged human p300, (**C**) histone octamers, and (**D**) mouse NAP1 and flag-tagged dACF/ISW1 complex.

**
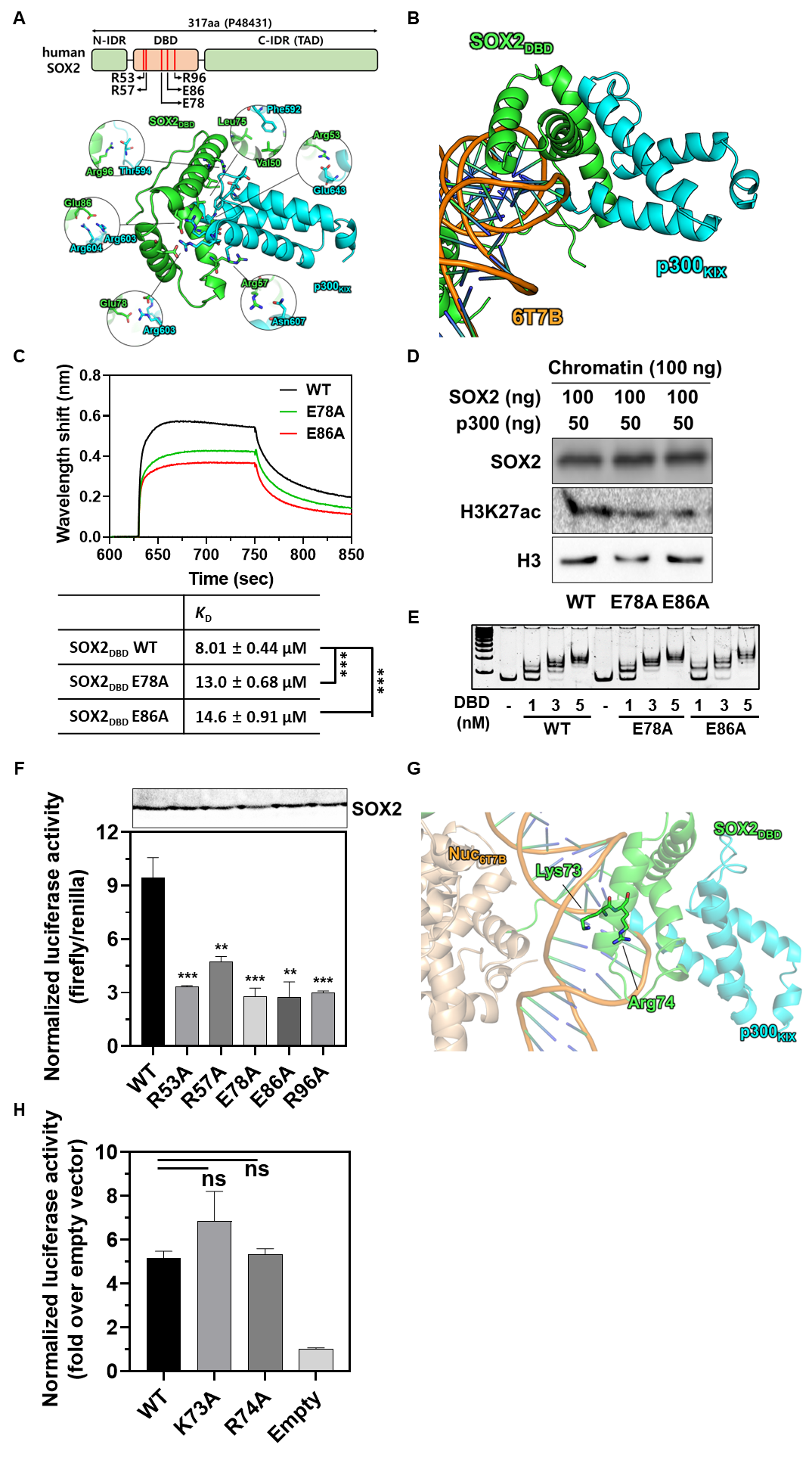
**

**Supplementary figure 9. Validation of SOX2:p300 interaction.** (**A**) Protein-protein docking simulation of SOX2_DBD_ (Green) and p300_KIX_ (Cyan). Structures of SOX2_DBD_ and p300_KIX_ were prepared by extracting the coordinates from the crystal structure of SOX2 (PDB:1GT0) and AlphaFold model of p300 (AF-Q09472-F1-model_v2), respectively. The simulation was performed using ClusPro server (20). The potent interacting residues are highlighted. The location of the potent interacting residues is labeled as a red bar in the schematic representation of SOX2. (**B**) The superposition of the docking model of SOX2_DBD_ (green):p300_KIX_ (cyan) on EM structure of SOX2_DBD_:nucleosome complex (PDB:6T7B). (**C**) BLI analysis of p300_KIX_ binding to wild type (WT, black) or mutants (E78A, green; E86A, red) SOX2_DBD_. Dissociation constant (*K*_D_) between SOX2_DBD_ and p300_KIX_ was calculated using association and dissociation rate (*k*_a_, *k*_d_) from the binding curve. (**D**) Western blot analysis of p300-mediated H3K27ac in the presence of WT or mutants SOX2. WT or mutants SOX2 (100 ng), p300 (50 ng), and chromatin of SM (100 ng) were incubated for 30 min at 30 °C. (**E**) EMSA of SOX2_DBD_ binding on SM. SM (1 nM) was incubated with different concentrations of WT or mutants SOX2_DBD_ for 1 h at room temperature before loading into the gel. (**F**) Luciferase activity from pGL3-SM in MEF cells with the overexpression of wild type (WT) or mutant (MT) SOX2. (**G**) Different angle of (Supplementary figure 9B) showing Lys73 and Arg74 of SOX2_DBD_. (**H**) Luciferase activity from pGL3-SM in MEF cells with overexpression of WT or mutant SOX2. All luciferase activities were normalized to Renilla luciferase activity from co-transfected pRL-TK plasmid, and their relative values to the activity of empty pGL3 reporter vector are shown. Error bars are standard error of the mean (SEM) of three independent experiments. *p*-values were calculated using a two-tailed t-test. (ns) *p* < 0.1234; (*) *p* < 0.0332; (**) *p* < 0.0021; (***) *p* < 0.0002; (****) *p* < 0.0001.

**
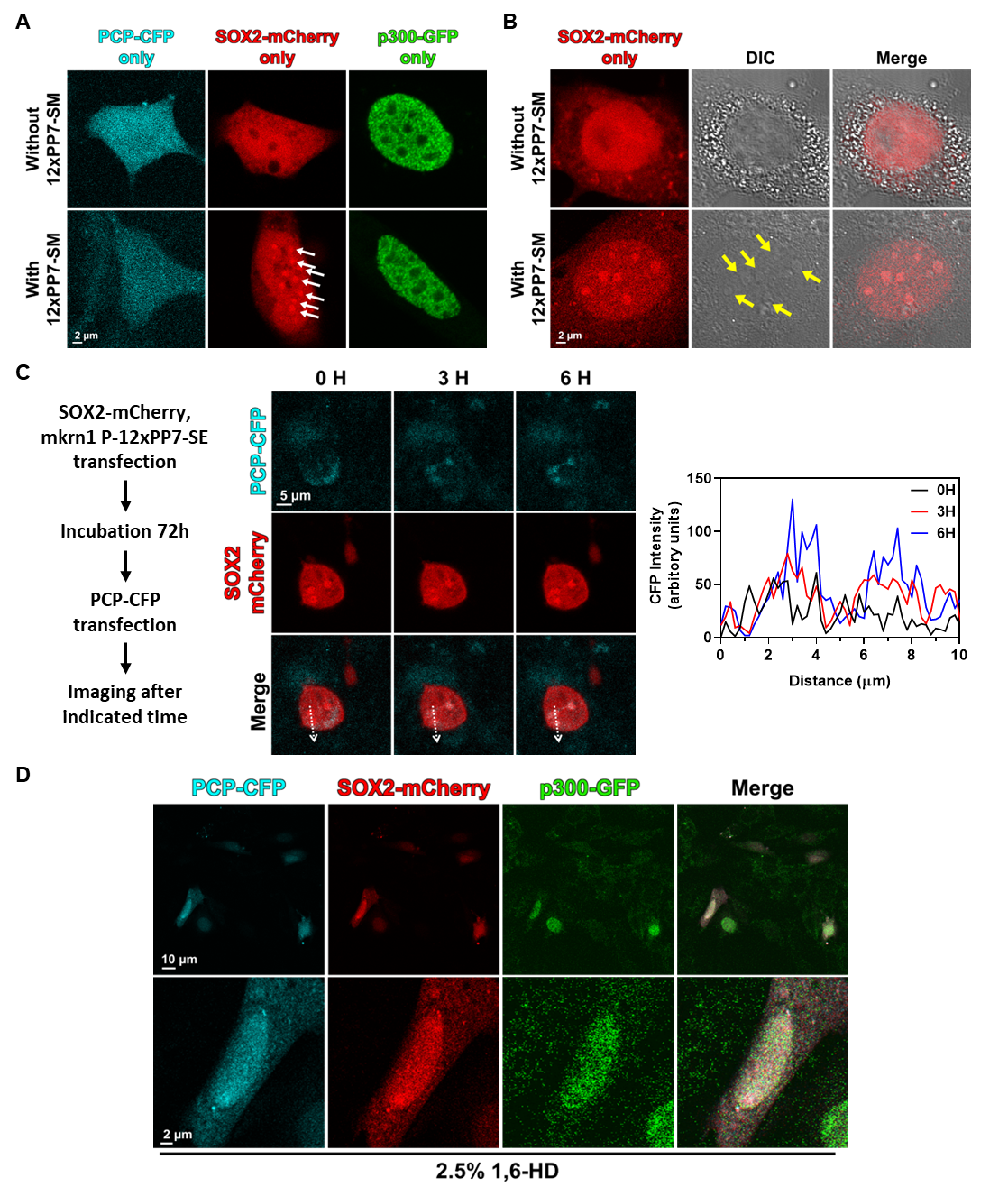
Supplementary Figure 10. Confocal microscopic analysis of PCP-PP7 system in the presence or absence of 1,6-hexandiol (1,6-HD). (A)** Confocal fluorescence and (**B**) differential interference contrast (DIC) images of MEF cells co-transfected with either tdPCP-CFP, SOX2-mCherry; or p300-GFP with or without 12xPP7-SM. Condensates in fluorescence and DIC image were highlighted by white and yellow arrows, respectively. (**C**) Left: time-course confocal analysis of MEF cells co-transfected with SOX2-mCherry, SM-containing reporter plasmid, and PCP-CFP. To see time-course co-condensation of CFP signals (PCP) on SOX2:SM condensates, PCP-CFP was transfected at 72-hour post-transfection of SOX2-mCherry and reporter plasmid. Right: line profiles of CFP fluorescence intensities of white line at 0, 3, and 6 h post-transfection of PCP-CFP. **(D)** Confocal microscopic analysis of tdPCP-CFP (transcript), SOX2-mCherry, and p300-GFP signals in MEF cells in the presence or absence of 2.5% 1,6-HD. In our condition, 1,6-HD above 2.5% was highly cytotoxic. Cells were imaged after 24 hours-post transfection. Top: 40X magnification images, bottom: closed-up images of single cells.

**
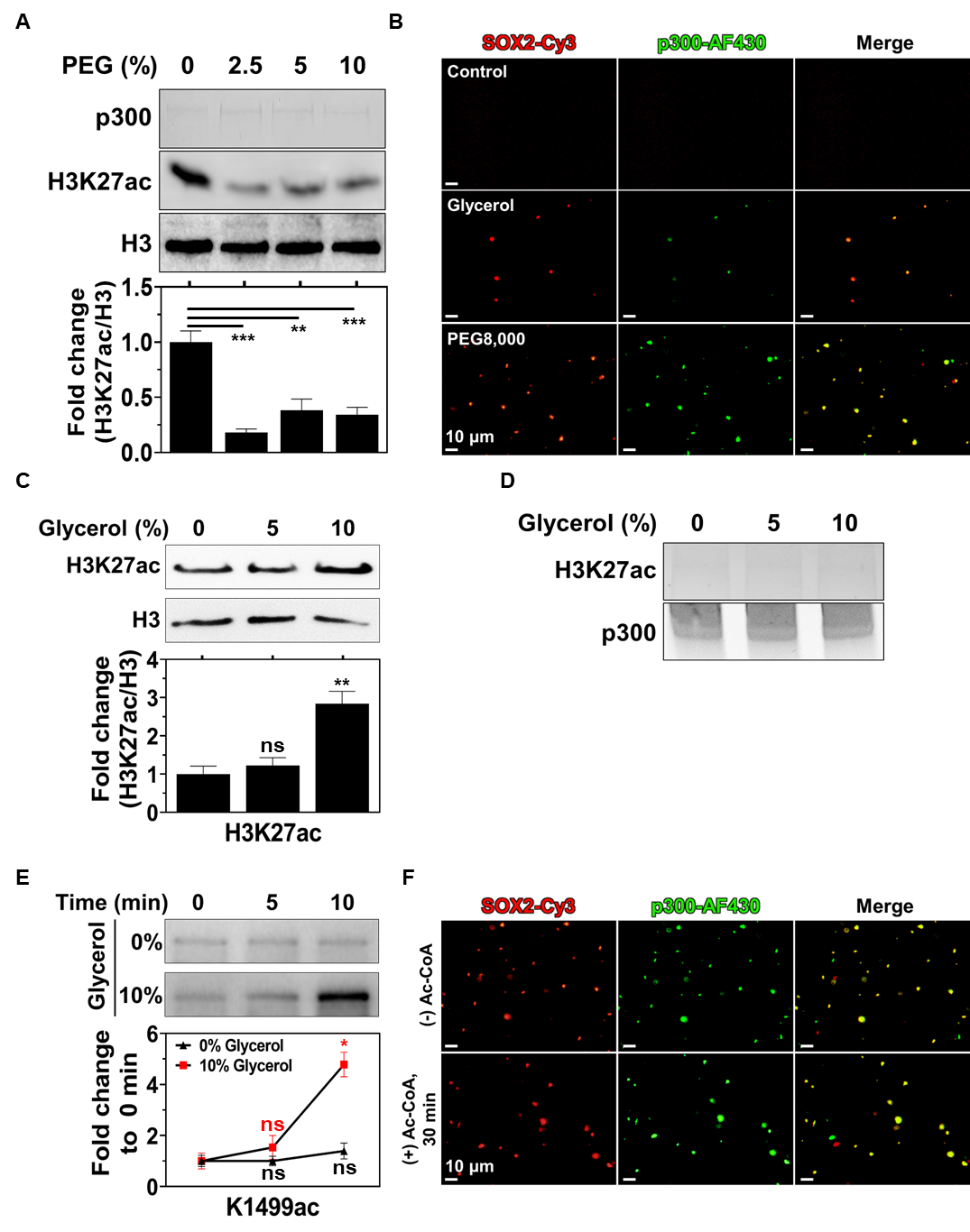
Supplementary Figure 11. *In-vitro* chromatin acetylation (IVA) assay in the presence of PEG8,000 or glycerol, p300 *trans*-autoacetylation in the presence of glycerol, and condensation of SOX2 and p300 in the presence of acetyl-CoA.** (**A**) Western blot analysis of IVA of histone octamers by p300 in the presence of different concentrations of PEG8,000. Band intensity was quantified in ImageLab software (Bio-Rad). Error bars are standard error of the mean (SEM) of three independent experiments. (**B**) Fluorescence (FS) microscopic analysis of co-localization of SOX2-Cy3 and p300-AF430 with chromatin of pGL3-SM in the presence of either 5% glycerol, 10% PEG8,000 or none (control). (**C**) Western blot analysis of p300-mediated H3K27ac in different glycerol concentrations in the presence of SOX2. (**D**) Western blot analysis of p300-mediated H3K27ac in different glycerol concentrations in the absence of SOX2. (**E**) Time-course analysis of SOX2-dependent *trans*-autoacetylation of p300 (K1499ac) in the absence or presence of glycerol. (**F**) FS microscopic analysis of co-localization of SOX2-Cy3 and p300-AF430 in IVA reaction mixture before and after adding acetyl-CoA. We observed no difference in SOX2:p300 co-condensation before and 30min-after treatment of acetyl-CoA, indicating that p300 acetylation does not affect SOX2:p300 co-condensation. *P*-values were calculated using a two-tailed *t*-test. (ns) *p* < 0.1234; (*) *p* < 0.0332; (**) *p* < 0.0021; (***) *p* < 0.0002.

**
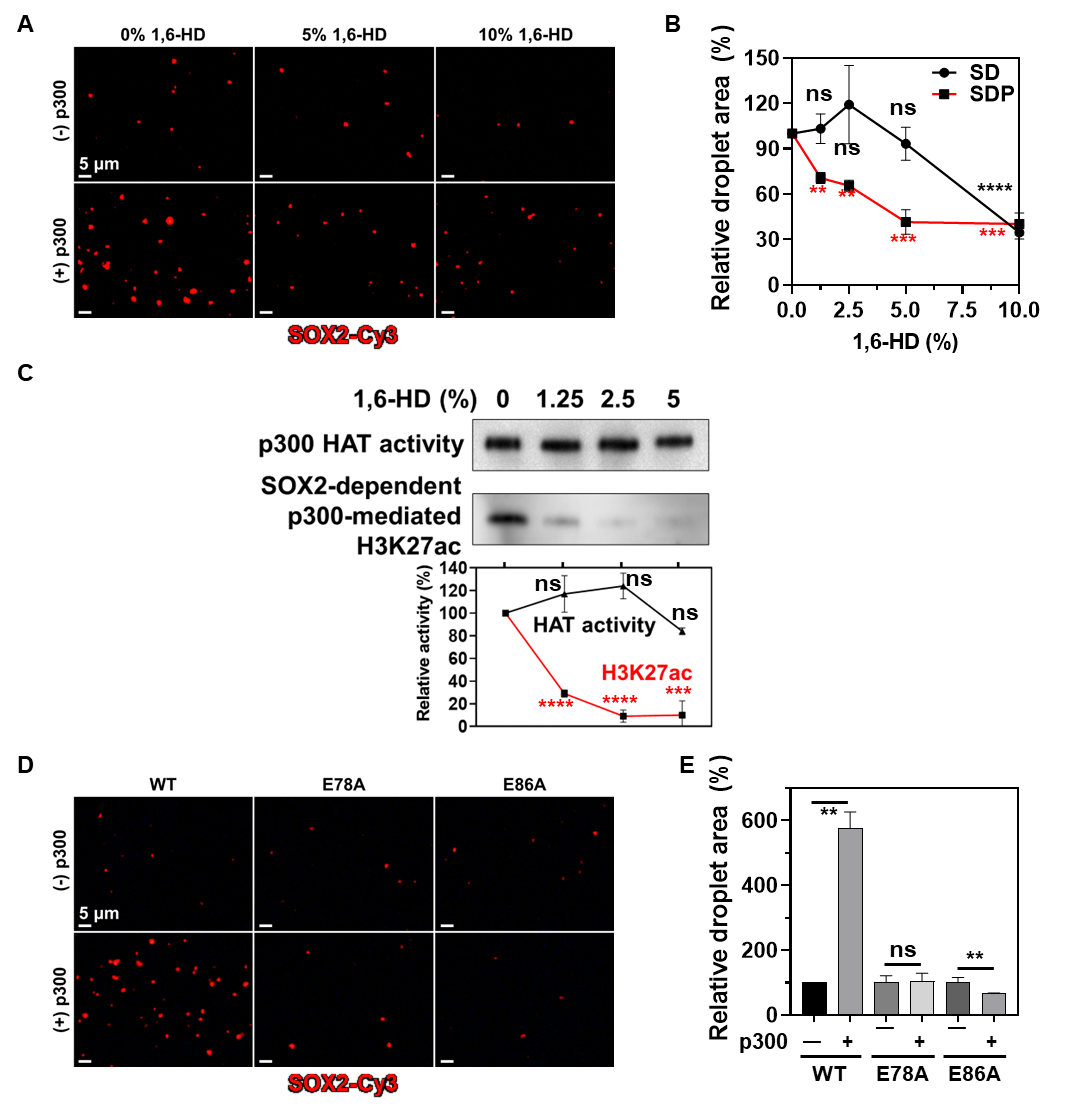
Supplementary Figure 12. Co-condensation of SOX2 and p300 and *in vitro* chromatin acetylation in the presence of 1,6-hexandiol (1,6-HD).** (**A**-**B**) Fluorescence (FS) microscopic analysis of SOX2 condensation in the presence of 1,6-hexandiol (1,6-HD). SOX2-Cy3 (837 nM) and SM (162 nM) were incubated with (+p300, SDP) or without (-p300, SD) p300 (126 nM) in different concentrations of 1,6-HD for 1 h at room temperature before imaging. The total area of droplets was quantified as described in the materials and methods section. Relative values to the total area of droplets with 0% 1,6-HD are shown. (**C**) Western blot analysis of p300 activity toward free histone octamer (HAT activity) and chromatins (SOX2-dependent p300-mediated chromatin acetylation) in different concentrations of 1,6-HD. Band intensity was quantified in ImageLab software (Bio-Rad). (**D**–**E**) FS microscopic analysis of condensation of WT or SOX2 mutants (E78A and E86A) with SM in the presence or absence of p300. SOX2-Cy3 (837 nM) and SM (162 nM) with or without p300 (126 nM) were incubated for 1 h at room temperature before imaging. The total area of droplets was quantified as described in the materials and methods section. Relative values to the sample containing WT SOX2, SM, and p300 are shown. Error bars are standard error of the mean (SEM) of at least three independent experiments. *P*-values were calculated using a two-tailed *t*-test. (ns) *p* < 0.1234; (*) *p* < 0.0332; (**) *p* < 0.0021; (***) *p* < 0.0002; (****) *p* < 0.0001.

**
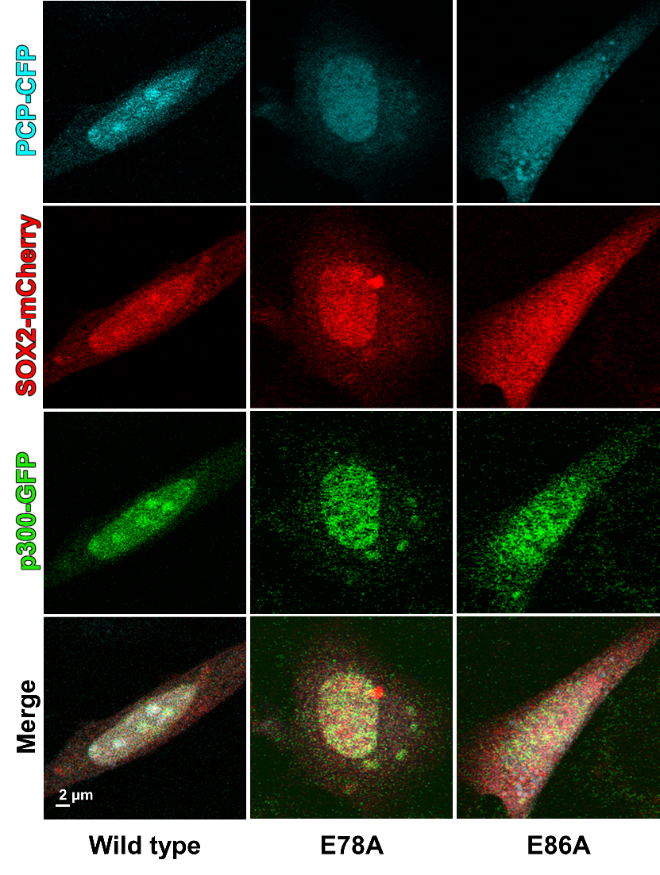
Supplementary Figure 13. Confocal microscopic analysis of PCP-PP7 system in MEF cells with wild type (WT) or mutants SOX2.** Confocal microscopic analysis of tdPCP-CFP (transcript), SOX2-mCherry, and p300-GFP signals in MEF cells with wild type (WT), E78A, or E86A SOX2 mutants. Cells were imaged after 24 hours-post transfection.

**
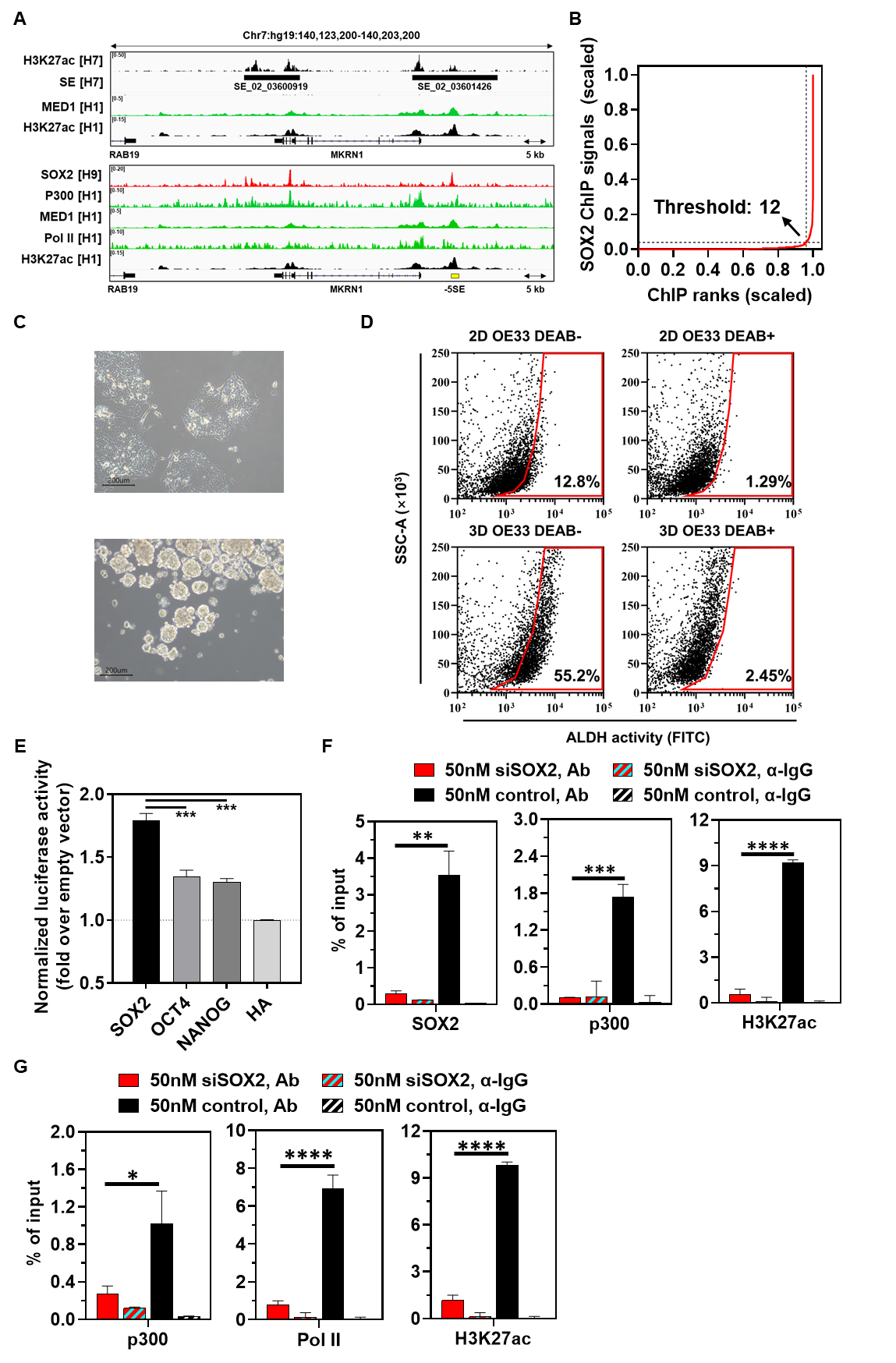
**

**Supplementary Figure 14. Genomic analysis near *MKRN1* TSS in human ESCs, fluorescence-activated cell sorting (FACS) analysis of OE33 cells, and luciferase assay of pGL3-(-5SE) in HEK cells with the expression of SOX2, OCT4 or NANOG.** (**A**) ChIP-seq profiles of SOX2, MED1, and H3K27ac near *MKRN1* gene in hESCs. ChIP-seq data for p300 and Pol II in hESCs were downloaded from Integrative Genomics Viewer (36). BED files for enhancer annotations were downloaded from reference (81). Images were rendered using Integrative Genomics Viewer. The location of -5SE is highlighted as yellow bar. (**B**) A plot of SOX2 ChIP-seq reads in KYSE-70 cells to a ranked order of reads by increasing value. The x and y axis were scaled from 0–1. A point (12) at which the slope of tangent line is 1 in the scaled plot was set as a threshold. (**C**) Differential interference contrast (DIC) images of 2D- (top) and 3D-cultured (bottom) OE33 cells. (**D**) FACS analysis of 2D- and 3D-cultured OE33 cells with ALDEFLUOR kit (StemCell Technologies, Vancouver, BC, Canada) using BODIPY-aminoacetaldehyde (BAAA) as a substrate. Diethylaminobenzaldehyde (DEAB) containing samples were used as negative controls. (**E**) Luciferase activity from pGL3-(-5SE) in HEK cells with the overexpression of either SOX2, OCT4, or NANOG. pcDNA3.1-HA was used as negative control. All luciferase activities were normalized to Renilla luciferase activity from co-transfected pRL-TK plasmid, and their relative values to the activity of empty pGL3 reporter vector are shown. (**F**) Chromatin immunoprecipitation followed by qPCR analysis to -5SE in OE33 cells. (**G**) Chromatin immunoprecipitation followed by qPCR analysis to MKRN1 promoter in OE33 cells. *P*-values were calculated using a two-tailed *t*-test. (ns) *p* < 0.1234; (*) *p* < 0.0332; (**) *p* < 0.0021; (***) *p* < 0.0002; (****) *p* < 0.0001.


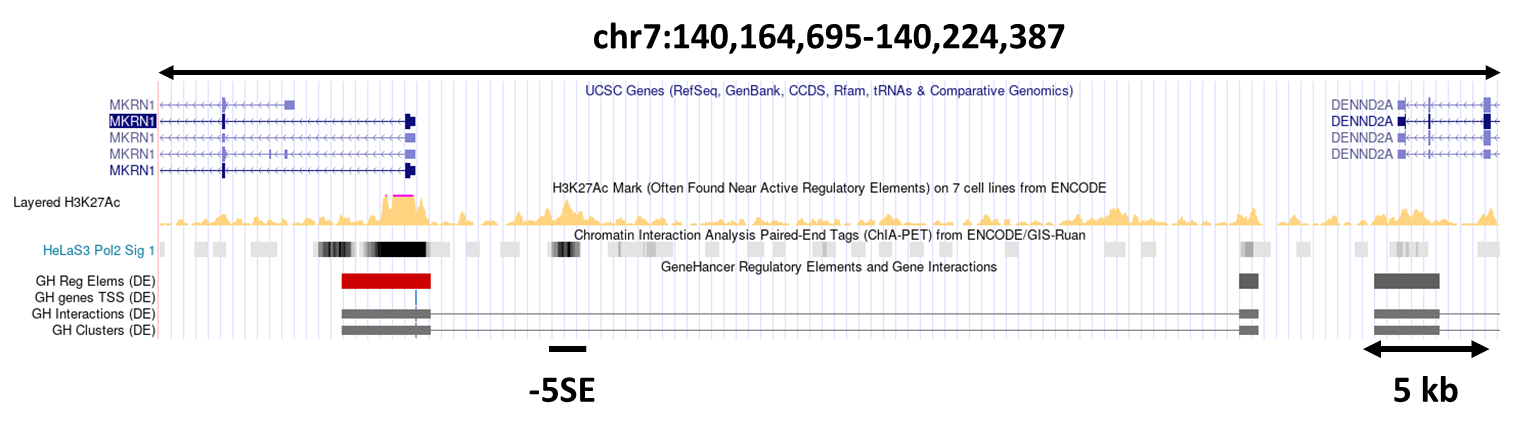
**Supplementary Figure 15. GeneHancer analysis of *MKRN1* TSS site in human ESCs.** H3K27ac in H1-hESCs (yellow track), ChIA-PET analysis of RNA Pol II in HeLa S3 cells (black dense map), and GeneHancer analysis results (referred as GH) are shown. Image was created by using UCSC genome browser.

**Supplementary Table 1. Primers used in this study**.

| **Primers** | **Sequences (5` to 3`)** |
| --- | --- |
| **Primers for cloning genes into pVFT1S vector** | |
| SOX2 forward | GAGCTGCGCTAGCATGTACAATATGATG |
| SOX2 reverse | GTATAGCCTCGAGTCACATATGTGAGAG |
| DBD_SOX2_ forward | GAGCTGCGAATTCGACCGCGTCAAGCGG |
| DBD_SOX2_ reverse | GTATAGCCTCGAGTTACAGCGTGTACTT |
| CTD_SOX2_ forward | GAGCTGCGAATTCGGCGCGGGCGTGAAC |
| CTD_SOX2_ reverse | GTATAGCCTCGAGTCACATGTGTGAGAG |
| KIX_p300_ forward | ACATGCAGCTAGCGGAATACGGAAACAG |
| KIX_p300_ reverse | GGATACGCTCGAGTTATTCTAGTTCTTTCTG |
| BR_p300_ forward | ATATGCAGCTAGCCGTCAGGATCCTGAA |
| BR_p300_ reverse | AGATATTCTCGAGTTAGTACACCCGTGATGT |
| KBH_p300_ forward | ACATGCAGCTAGCGGAATACGGAAACAG |
| KBH_p300_ reverse | AGATATTCTCGAGTTATTGGCTCTGCGTGTG |
| **Primers for cloning genes into pcDNA3** | |
| SOX2 forward | ACATGCAGGTACCATGATGGAGACGGAG |
| SOX2 reverse | AGATATTCTCGAGTCACATGTGCGACAGGGG |
| OCT4 forward | ACTAGCAGGTACCATGGCTGGACATCTGGCT |
| OCT4 reverse | GAATCATCTCGAGTCAGTTTGAATGCATGGGGGAGCC |
| NANOG forward | ACTAGCAGGTACCATGATGAGTGTGGGAATT |
| NANOG reverse | AGCTATTCTCGAGTCATATTTCAATTGTAGGAGTCAC |
| **Primers for amplification** | |
| SM forward | GCGGTCCATTATTCC |
| SM reverse | GTAGTCCTTGCATGT |
| NM forward | GGGTCGGACCCTCTG |
| NM reverse | GTAGTCCTTGCATGT |
| **Primers for site-directed mutagenesis and RT-PCR** | |
| dSM forward | GAATATCTTCTTATCTTGGGGCCGCAC |
| dSM reverse | GTGCGGCCCCAAGATAAGAAGATATTC |
| rNM forward | GGTGGCTCACAAAAGTCTATAATGGGA |
| rNM reverse | TCCCATTATAGACTTTTGTGAGCCACC |
| SOX2 R53A forward | ATGGTATGGTCCGCGGGGCAGCGGCGT |
| SOX2 R53A reverse | ACGCCGCTGCCCCGCGGACCATACCAT |
| SOX2 R57A forward | CGGGGGCAGCGGGCGAAGATGGCCCAG |
| SOX2 R57A reverse | CTGGGCCATCTTCGCCCGCTGCCCCCG |
| SOX2 E78A forward | CGCCTGGGCGCGGCGTGGAAACTTTTG |
| SOX2 E78A reverse | CAAAAGTTTCCACGCCGCGCCCAGGCG |
| SOX2 E86A forward | TTGTCCGAGACCGCGAAGCGGCCGTTC |
| SOX2 E86A reverse | GAACGGCCGCTTCGCGGTCTCGGACAA |
| SOX2 E95A forward | CCGTTCATCGACGCGGCCAAGCGGCTG |
| SOX2 E95A reverse | CAGCCGCTTGGCCGCGTCGATGAACGG |
| SOX2 forward | GCTACAGCATGATGCAGGACCA |
| SOX2 reverse | TCTGCGAGCTGGTCATGGAGTT |
| MKRN1 forward | TTCCAAGTGAGTACTGG-GTGGA |
| MKRN1 reverse | AAACAGTTCCCTCCAAATGGGC |
| β-actin forward | TGACGGCCAGGTCATCACCATT |
| β-actin reverse | AATGCCAGGGTACATGGTGGTG |
| **Primers for ChIP-qPCR** | |
| mSE078 forward | TAGTCGAGCTCTGCCGCTAA |
| mSE078 reverse | CTGGCTCAACCAGGAAATCG |
| Continued. |  |
| **Supplementary Table 1. Continued.** | |
| mSE191 forward | GCAAGATCCCCACGTGACAA |
| mSE191 reverse | TATTGCGTCATCACTAGCTTCCA |
| mSE219 forward | AGCAGTTCAGAGAACCAGGC |
| mSE219 reverse | TGCAAATGTCCAGGAGAGGG |
| mSE224 forward | TTTGCTGCTGAAGGGTAGGG |
| mSE224 reverse | CCTACTTTCGGACCTCGTCG |
| mSE227 forward | GTGTTAACAGCTGGGAGGGA |
| mSE227 reverse | GGCGCCTTGTGAAAGTCTTC |
| -5SE forward | GGGCACTTTTGTTCCTGCAC |
| -5SE reverse | GGGCACTTTTGTTCCTGCAC |
| MKRN1 promoter foward | AAGGACCACATGCAAGGACC |
| MKRN1 promoter reverse | AAGGACCACATGCAAGGACC |

**Supplementary Table 2. Kinetic parameters of the binding of SOX2 and p300 domains.**

**^a^** Equilibrium binding constant (*K*_D_), association rate (*k*_a_) and dissociation rate (*k*_d_) of were calculated by globally fitting the curve to 1:1 binding model. The fitting was performed using BLItz Pro v.1.2.1.5 software.

|  | ***K*_D_ (M)^a^** | ***k*a (M^-1^ s^-1^)^a^** | ***k*d (s^-1^)^a^** | **R^2^** |
| --- | --- | --- | --- | --- |
| SOX2-KIX | 2.68E-06 | 1.23E+04 ± 6.98E+02 | 4.69E-02 ± 8.63E-04 | 0.96 |
| SOX2-BD | 5.40E-05 | 1.12E+02 ± 1.79E+01 | 6.06E-03 ± 2.77E-04 | 0.85 |
| SOX2-KB | 6.47E-08 | 6.36E+03 ± 4.56E+02 | 4.12E-03 ± 6.07E-05 | 0.99 |
| SOX2-BSA | n.d. | n.d. | n.d. | n.d. |

**Supplementary Table 3. Kinetic parameters of the binding of p300 domains to SOX2_DBD_ or SOX2_CTD_.**

|  | ***K*_D_ (M^-1^)^a^** | ***k*a (M^-1^ s^-1^)^a^** | ***k*d (s^-1^)^a^** | **R^2^** |
| --- | --- | --- | --- | --- |
| SOX2_DBD_-KIX | 9.27E-06 | 5.06E+03 ± 1.95E+02 | 4.69E-02 ± 1.28E-03 | 0.96 |
| SOX2_DBD_-BD | 1.54E-04 | 1.72E+02 ± 4.62E+01 | 2.65E-02 ± 6.86E-04 | 0.95 |
| SOX2_CTD_-KIX | 4.93E-05 | 1.72E+03 ± 1.39E+02 | 8.49E-02 ± 2.30E-03 | 0.96 |
| SOX2_CTD_-BD | 2.64E-01 | 3.60E-01 ± 3.97E+02 | 9.51E-02 ± 1.28E-02 | 0.67 |

**^a^** Equilibrium binding constant (*K*_D_), association rate (*k*_a_) and dissociation rate (*k*_d_) of were calculated by globally fitting the curve to 1:1 binding model. The fitting was performed using BLItz Pro v.1.2.1.5 software.
